# To Predict Anti-Inflammatory and Immunomodulatory Targets of Guizhi Decoction in Treating Asthma Based on Network Pharmacology, Molecular Docking, and Experimental Validation

**DOI:** 10.1155/2021/9033842

**Published:** 2021-12-20

**Authors:** Rui Sun, Gonghao Xu, Dongyang Gao, Qi Ding, Yuanyuan Shi

**Affiliations:** ^1^School of Life Sciences, Beijing University of Chinese Medicine, Beijing 100029, China; ^2^Shenzhen Research Institute, Beijing University of Chinese Medicine, Shenzhen 518118, China; ^3^School of Chinese Materia Medica, Beijing University of Chinese Medicine, Beijing 100029, China

## Abstract

Asthma, characterized by the continuous inflammatory response caused by a variety of immune cells, is one of the most common chronic respiratory diseases worldwide. Relevant clinical trials proved that the traditional Chinese medicine formula Guizhi Decoction (GZD) had multitarget and multichannel functions, which might be an effective drug for asthma. However, the effective ingredients and mechanisms of GZD against asthma are still unclear. Therefore, network pharmacology, molecular docking, and cell experiments were performed to explore the antiasthma effects and potential mechanisms of GZD. First, we applied the TCMSP database and literature to obtain the bioactivated ingredients in GZD. SwissTargetPrediction, TCMSP, GeneCards, OMIM, PharmGkb, TTD, DrugBank, and STRING database were used to get core genes. In addition, the key pathways were analyzed by the DAVID database. Molecular docking was used to predict whether the important components could act on the core target proteins directly. Finally, qPCR was carried out to verify the network pharmacology results and the possible mechanisms of GZD in the treatment of asthma. We collected 134 active ingredients in GZD, 959 drug targets, and 3223 disease targets. 431 intersection genes were screened for subsequent analysis. Through GO and KEGG analyses, enriched pathways related to inflammation and immune regulation were presented. Through the qPCR method to verify the role of essential genes, we found that GZD had an excellent anti-inflammatory effect. Direct or indirect inhibition of MAPK and NF-*κ*B pathways might be one of the crucial mechanisms of GZD against asthma. GZD might be a promising potential drug for the treatment of asthma. This article provided a reference for the clinical application of GZD.

## 1. Introduction

Asthma, a common chronic inflammatory respiratory disease globally, is characterized by wheezing, chest tightness, shortness of breath, and cough in clinical [1]. There are nearly 300 million asthmatics worldwide. Patients will also have complications such as respiratory failure, cardiovascular disease, and kidney disease, which reduces the patient's quality of life and increases the economic burden [1, 2]. Asthma might be induced by a combination of genetic and environmental factors [3]. Main pathological manifestations include bronchoconstriction, airway inflammation, and airway hyperresponsiveness (AHR) [1, 4]. The pathological mechanism of asthma has not been fully elucidated, but it is known that inflammation and immune regulation play a vital role in the occurrence and development of asthma [4]. At present, although anti-inflammatory and bronchodilator treatment strategies are used frequently in clinical, problems such as treatment tolerance and side effects are still prominent. So, new effective therapeutic drugs with less side effects are in urgent need of development [5].

With the continuous development of Chinese medicine research, traditional Chinese medicine (TCM), due to its therapeutic efficacy and few side effects, is quickly accepted by clinicians in China [6, 7]. Guizhi Decoction (GZD), derived from “Treatise on Febrile and Miscellaneous Diseases” by Zhang Zhongjing, a medical sage of the Han Dynasty, is the most widely used, the most frequently used, and the most widely derived decoction. As a well-known superficies-relieving formula, GZD has the effects of relieving exterior syndrome with pungent and warmth, relieving muscles and sweating, reconciling Ying and Wei, which is usually applied to the cold caused by the disharmony of Ying and Wei [8, 9]. Previous studies have shown that GZD and its addition and subtraction formulations are often used in clinical treatment for asthma patients. Mechanisms of GZD against asthma may inhibit the TGF-*β*1/Smad2 pathway, NF-*κ*B pathway, and TLR4 pathway by reducing the expression of inflammatory factors [10–14]. Moreover, researchers studied the clinical application of GZD and Yupingfeng Powder on asthma [15–17]. Due to the complex components of GZD and the unclear pathological mechanism of asthma, we used network pharmacology, molecular docking combined with experimental validation to explain the therapeutic effect of GZD on asthma in this study.

**Figure 1:**
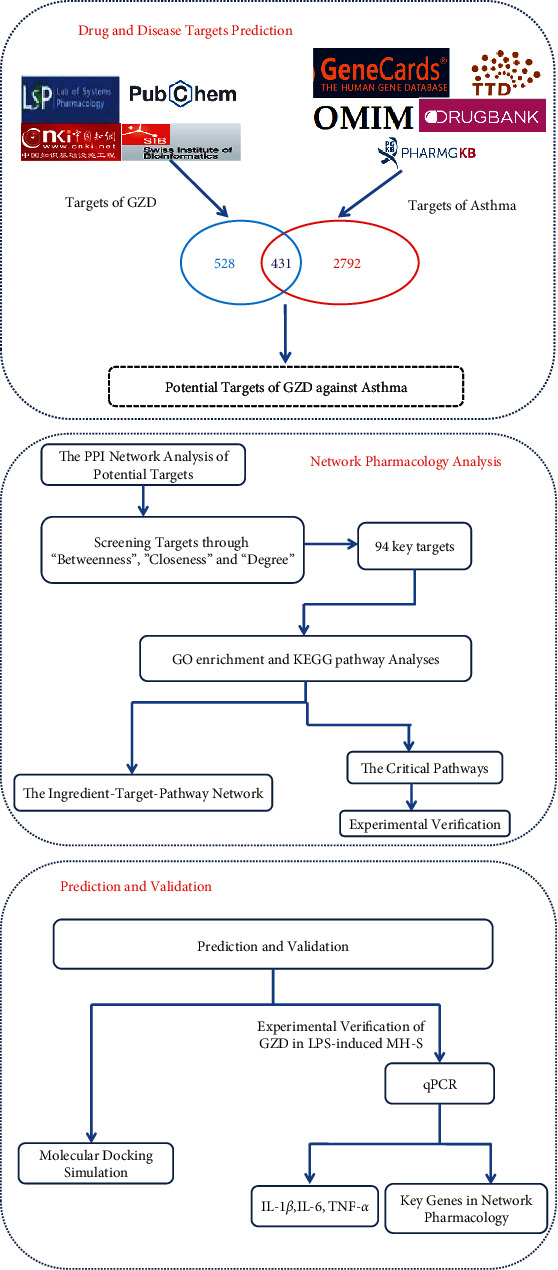
Graphical abstract. Network pharmacology, molecular docking, and experimental verification methods were used to clarify the pharmacological mechanism of GZD against asthma.

Network pharmacology, based on network colossal data analysis, can predict the potential mechanism of traditional Chinese medicine and compound prescriptions scientifically and gradually become a powerful tool to accelerate the modernization of traditional Chinese medicine. A growing number of researchers are applying network pharmacology methods to explore the mechanism of actions of Chinese medicine in the treatment of related diseases [1, 18, 19]. Here, we used the method of network pharmacology and molecular docking to predict the mechanism of GZD in the treatment of asthma and analyzed potential active ingredients, targets of GZD and asthma, PPI, and pathway. Subsequently, experiments in vitro verified the predicted results of network pharmacology (Figure 1). This study provides new ideas for the treatment of asthma through network pharmacology and provides a basis for the clinical application of GZD for asthma, which is of great significance for the development of compound preparations.

## 2. Materials and Methods

### 2.1. Collection of Potential Targets of GZD

The five traditional Chinese medicines of GZD, namely, Guizhi (*Cinnamomum cassia)*, Shaoyao *(Paeonia lactiflora)*, Gancao *(Glycyrrhiza uralensis)*, Shengjiang (*Zingiber officinale*), and Dazao (*Ziziphus jujube*), were used as keywords for screening active ingredients in the system pharmacology database and analysis platform of Chinese medicine (TCMSP: https://tcmsp-e.com/). TCMSP is one of the world's largest noncommercial TCM molecular databases. It collects 499 herbs and the compound components of each herb from the 2010 Chinese Pharmacopoeia, including 837 diseases and 29,384 compounds. In addition, TCMSP also provides comprehensive data on absorption, distribution, metabolism, and excretion characteristics of each compound, including drug similarity (DL) and oral bioavailability (OB). OB ≥ 30% and DL ≥ 0.18 are the most critical indicators for screening vital active compounds. We used “guizhi,” “baishao,” “gancao,” “shengjiang,” and “dazao” as keywords, respectively, and imported them into TCMSP to search and screen vital active compounds. After the initial screening, the compounds were checked one by one or supplemented by searching the literature to get the final active compound list. The SDF format files of GZD compounds were derived from PubChem (put the chemical components in GZD into the PubChem database to get their chemical structures and download their SDF format for the two-dimensional structures). Then, the SwissTargetPredicition platform was used (http://www.swisstargetprediction.ch/) to predict compound targets (put its two-dimensional structure into SwissTargetPrediction to predict its drug targets). For compounds that were not available on the SwissTargetPrediction website, we searched in TCMSP and GeneCards.

### 2.2. Collection of Potential Targets against Asthma

Asthma is mainly manifested as tachypnea, wheezing, and coughing. To obtain relevant targets, we searched five databases separately, such as GeneCards (https://www.genecards.org), OMIM (https://www.omim.org/), PharmGkb (https://www.pharmgkb.org/), TTD (http://db.idrblab.net/ttd/) and DrugBank (https://go.drugbank.com/). “Asthma” was input into databases as the keyword for obtaining targets of asthma. After merging results, duplicates were removed to obtain the disease targets group. Finally, the intersection network of compound-target and disease-target was analyzed using the Draw Venn Diagram, which represented potential targets of GZD against asthma.

### 2.3. Protein-Protein Interaction (PPI) Network Construction

The STRING database is a robust database to predict the interactions between proteins. For further analyses, the STRING database (https://string-db.org/) was used to construct a potential target PPI network. The number of nodes, edges, and the PPI enrichment value could be evaluated in the STRING database. “Homo sapiens” and high confidence 0.9 were selected as the confidence interval for protein interactions. After evaluating the topological importance of nodes by calculating three topological features as “betweenness”, “closeness”, and “degree”, essential targets were screened out in the network. We calculated the median, respectively, and selected the genes whose topological feature values were all greater than the median as the core genes (chose the genes that satisfied betweenness > 0, closeness > 0.400862069, and degree > 3). Visual analysis was performed in the Cytoscape 3.7.2 software.

### 2.4. Gene Ontology (GO) and Kyoto Encyclopedia of Genes and Genomes (KEGG) Signaling Pathway Analysis

GO is an analysis widely used to classify gene functions and describe the functions of gene products. GO analysis includes a biological process (BP), cellular component (CC), and molecular function (MF). BP describes the physiological or cellular functions of the selected genes. KEGG pathway analysis is a knowledge base for systematic gene function analysis, linking genomic information with practical information to obtain significant biological pathways. The list of core targets obtained in Section 2.3 was entered into DAVID 6.8 (https://david.ncifcrf.gov/tools.jsp) for GO and pathway analysis. *pvalue* <0.05 was considered statistically significant. Finally, the visualization of the bubble chart was realized by using “R”, and the “Ingredient-target-pathway” network was performed in the Cytoscape 3.7.2 software.

### 2.5. Molecular Docking Simulation

In order to evaluate the credibility of the connection between the core targets with the vital compounds and predict new candidate compounds for the treatment of asthma, so we docked the vital compounds with the core target proteins. The nine highest-degree targets in the “Ingredient-target-pathway” network were used as receptors, and five highest-degree compounds were considered ligands. In addition, in order to evaluate the results of molecular docking, we selected the known ligands of the top nine targets to dock with its target as the longitudinal positive control. And we selected the clinical treatment of asthma drug methylprednisolone and its known ligands NR3C1 [20] as the horizontal positive control. First, we put the small molecule ligands 2D structure obtained from the PubChem website into Chem3D software to convert small molecule ligands into 3D structures and then utilized PDB web (https://www.rcsb.org/) to obtain the two-dimensional structure of the protein receptor, after which we used PyMOL software to delete water molecules and small-molecule ligands. Next, AutoDock Vina software was employed to convert protein receptors and small molecule ligands into PDBQT format. By searching for the location of the known ligands of protein receptors on the PDB web, we determined the location of the protein receptor's functional pockets to prepare for molecular docking. Then, PyMOL software was used to visualize the docking situation. Finally, we displayed the interaction force between each chemical bond by LigPlot software and used Vina to evaluate the binding energy that showed the binding strength between the ligand and the target protein. Binding energy is considered to be the most essential indicator for judging the results of docking [21]. The smaller the binding energy, the more stable the binding, the binding energy < -5.00 kcal/mol indicates good binding strength, and the binding energy < -7.00 kcal/mol indicates sufficient binding strength [22]. PyMOL and LigPlot were used to analyze the docking conformation visually. From the binding conformations of the docking results of each compound, results with lower binding energy were selected for display. When the binding energies were consistent, we preferred to display the conformation containing hydrogen bonds, which would be more stable.

### 2.6. Experimental Validation

#### 2.6.1. Cell Culture

The mouse alveolar macrophage MH-S cell line was purchased from Being BioTech (China) and was maintained in RPMI-1640 medium containing 10% FBS at 37°C in 5% CO_2_ humidified air. We changed the culture medium of the cells every other day, and when the cells were grown to the logarithmic growth phase, we passed them down.

#### 2.6.2. Drug Preparation

Traditional Chinese medicine was purchased from Tongrentang Pharmacy. According to the ratio of Guizhi: Shaoyao: Shengjiang: Gancao: Dazao = 9 : 9 : 9 : 6 : 3 pieces, a total of 27.4561 g of traditional Chinese medicine materials was used; five times the amount of 75% ethanol was soaked for 2 hours and then extracted for 1 hour by the water bath. The extract was collected and then extracted for the second time. The two extracts were combined and concentrated. The concentrated solution was ultrasonically extracted with 20 times absolute ethanol, and the macromolecules were removed. Finally, 3.7718 g of concentrated liquid was obtained, and the yield was 13.74%. The concentrated solution was prepared into a 500 mg/mL (equivalent amount of crude drug) stock solution, and it was kept in a refrigerator at 4°C for later use. We configured it into a final drug concentration of 1, 10, 100, 200, and 500 *μ*g/mL with DMSO before the experiment.

#### 2.6.3. CCK-8 and Drug Administration

A cell counting Kit-8 (CCK-8, Jinpulai, China) assay was performed to assess the viability of MH-S cells after GZD treatment. Briefly, the cells were seeded into 96-well plates at a density of 8 × 10^3^ cells/200 *μ*L after culturing for 4 hours without serum and then treated with GZD (0, 1, 10, 100, 200, and 500 *μ*g/mL) for 24 h. Next, 20 *μ*L CCK-8 solution was added to each well. Then, the plates were incubated at 37°C for another 2.5 h. Finally, the optical density of each well was measured at 450 nm using a microplate reader. Then, we stimulated MH-S cells with lipopolysaccharide (LPS) to construct an in vitro inflammation model, took the MH-S cells in the logarithmic growth phase, planted the cells in a 96-well plate at 8 × 10^3^ cells/200 *μ*L, cultured them for 4 hours without serum, and then administered and added LPS (final concentration of 5 *μ*g/mL) for 24 hours [23–26]. The concentration setting and methods were the same as above.

#### 2.6.4. Quantitative Real-Time PCR (qPCR)

We planted cells in six-well plates at 1 × 10^6^ cells/2 mL for mRNA extraction. For each experiment, the cells were stimulated with LPS (5 *μ*g/mL) in the presence or absence of GZD (0, 10, 100, and 200 *μ*g/mL) for 24 h. Total RNA was extracted with an RNA extraction kit (Tian Gen, China) from the MH-S cells, and RNA concentration was measured before reverse transcription (HiScript II Q RT SuperMix, Vazyme, China). Total RNA (2000 ng) was further reverse transcribed into cDNA. With glyceraldehyde-3-phosphate dehydrogenase (GAPDH) as the endogenous control, then quantified with SYBR (ChamQ SYBR qPCR Master Mix, Vazyme, China), the relative expression level was calculated using the comparative threshold cycle (2−ΔΔCT) equation. The primer details are shown in Table 1.

### 2.7. Statistical Analysis

The SPSS 21.0 software and GraphPad Prism 6 were used for statistical analysis of the data. The paired sample *T*-test was applied for comparison between two samples, and the analysis of variance was used for multiple sample comparisons. *p* < 0.05 indicated that the difference was statistically significant.

## 3. Results

### 3.1. Active Ingredients of GZD and Their Potential Targets

In this study, 134 compounds including quercetin and paeoniflorin were obtained from TCMSP or literature research (refer to Section 2.1 for specific methods). Detailed information about compounds was performed in Supplementary Material Table S1 (134 active compounds from TCMSP database and literature in Guizhi Decoction). After removing the duplicate values, a total of 959 drug targets were obtained from SwissTargetPrediction, TCMSP, and GeneCards. Detailed information on relevant drug targets was performed in Supplementary Material Table S2 (drug targets information of different ingredients in Guizhi Decoction).

### 3.2. Screening of Asthma Targets

**Figure 2:**
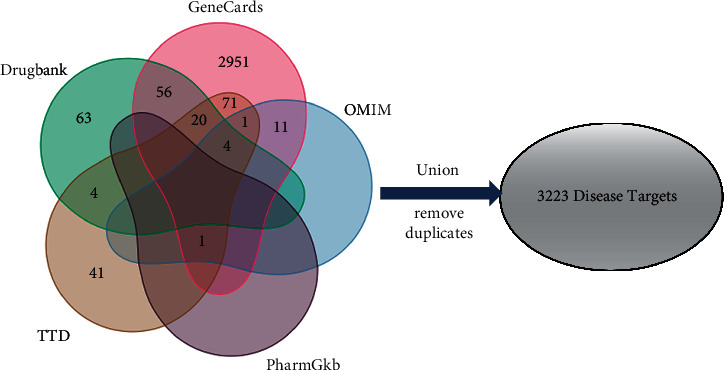
Screening of asthma targets. Venn diagram of targets for asthma from DrugBank (green), GeneCards (pink), OMIM (blue), PharmGkb (purple), and TTD (orange) databases.

407, 3115, 17, 1, and 146 disease targets were, respectively, obtained from DrugBank, GeneCards, OMIM, PharmGkb, and TTD. Their relationships were visualized with Venn diagrams. After removing duplicate values, a total of 3223 asthma-related targets were obtained (Figure 2).

### 3.3. PPI Network Construction and Core Target Selection

**Figure 3:**
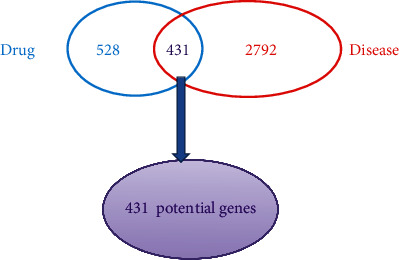
Venn diagram of the potential genes in asthma and GZD. The overlapped genes represented core genes (purple). The blue part represented the unique drug targets, and the red part represented the unique disease targets.

**Figure 4:**
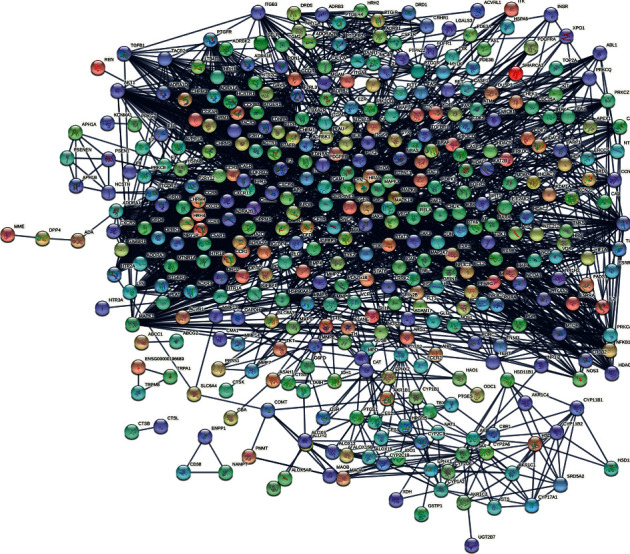
PPI network construction. We took the intersection of drug targets and disease targets to obtain 431 intersection genes of GZD against asthma and put them into STRING to analyze the correlation between them, and to build a PPI visualization network, high confidence 0.9 was selected as the confidence interval. The connection represented the correlation between protein and protein.

**Figure 5:**
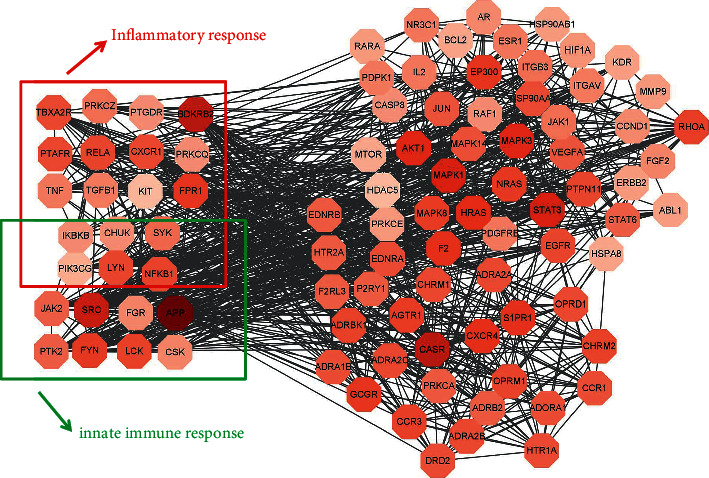
PPI network of core targets. As the degree increases, the color is darker (closer to red). Innate immune response-related genes are highlighted in the green box; inflammatory response-related genes are highlighted in the red boxes.

We took the intersection of drug targets and disease targets to obtain 431 intersection genes of GZD against asthma (Figure 3). After putting the genes into STRING and analyzing the correlation between them, we built a PPI visualization network (Figure 4). According to the distribution of the network degree, by filtering “betweenness”, “closeness”, and “degree”, respectively, we screened 94 core targets which were satisfied betweenness > 0, closeness > 0.400862069, and degree > 3. Detailed information of intersection genes and core targets was, respectively, performed in the Supplementary Material Table S3 (target information at the intersection of drug targets and disease targets) and Supplementary Material Table S4 (core gene information filtered according to the “betweenness”, “closeness”, and “degree” values). After putting 94 core genes into Cytoscape to visualize, we found that a majority of these genes were related to inflammation and immune-related diseases (Figure 5).

### 3.4. GO Analysis and KEGG Pathways Involved in the Treatment of Asthma with GZD

**Figure 6:**
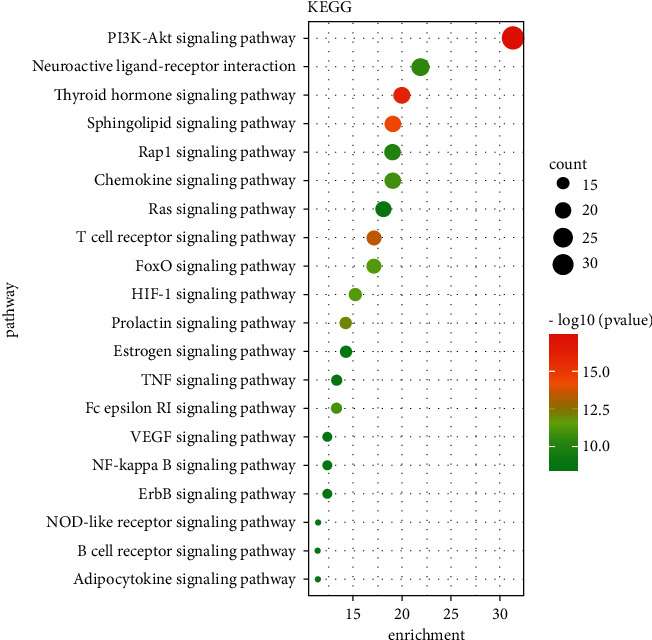
Bubble diagram of functional pathway analysis. Pathway enrichment analyses of 94 core targets by DAVID database. The size of the circle represented the number of genes; the shade of the color represented the size of the *p* value.

**Figure 7:**
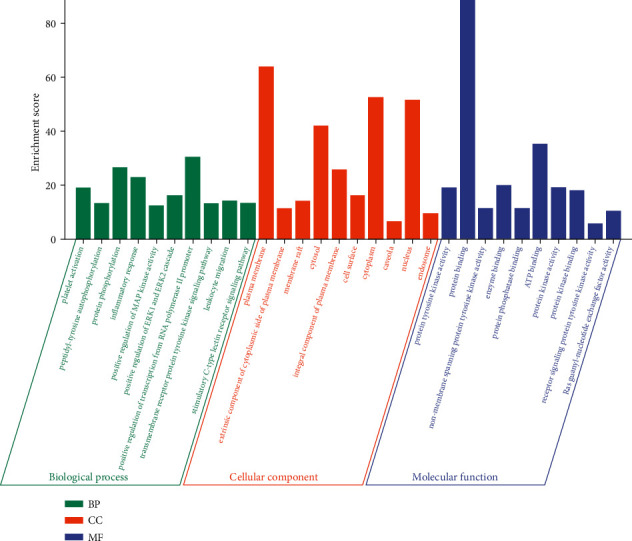
Histogram of GO analyses. GO enrichment analyses of 94 core targets by DAVID database. Green represented the biological process, orange represented the cellular component, and blue represented the molecular function.

According to the 94 core targets obtained in Section 3.3, the DAVID database was used for the enrichment analysis of GO and KEGG pathways (Figures 6 and 7). We screened the top of its BP, CC, MF, and pathways based on the *pvalue*. Among them, the key biological processes included inflammatory response, immune response, MAPK cascade, and response to lipopolysaccharide; the significant pathways could be divided into inflammation (e.g., NF-kappa B signaling pathway and TNF signaling pathway), immune response (e.g., T cell receptor signaling pathway and B cell receptor signaling pathway), and signal transduction (e.g., PI3K-Akt signaling pathway, MAPK signaling pathway, and RAS signaling pathway).

### 3.5. Ingredient-Target-Pathway Network Construction

**Figure 8:**
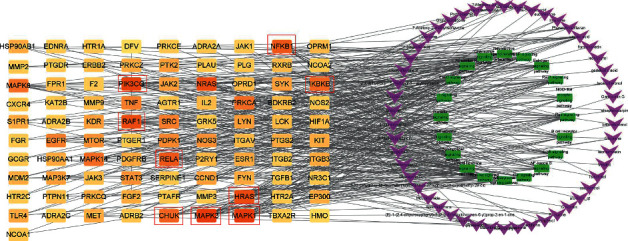
Ingredient-target-pathway network. We analyze the relationships of vital ingredients, key targets, and top 20 pathways. Purple “V” nodes represented the ingredients in GZD; orange nodes represented the key targets (as the degree increases, the color closed to orange; the targets in the red box were the focus of subsequent experimental validation); green nodes represented the top pathways.

Based on the key ingredients, core targets, and pathways obtained above, an integrated ingredient-target-pathway network was constructed by using Cytoscape 3.7.2 (Figure 8). The ingredient-target-pathway network consisted of 161 nodes and 430 edges. The nodes with a higher degree in the network were considered as vital targets. According to a degree, MAPK1, MAPK3, RAF1, NRAS, PIK3CG, NFKB1, IKBKB RELA, and CHUK were the first nine targets. These nodes also interacted closely with the NF-*κ*B signaling pathway and MAPK signaling pathway. Ursolic acid, quercetin, phaseolinisoflavan, moupinamide, and glabrene were the top five compounds.

### 3.6. Molecular Docking

**Figure 9:**
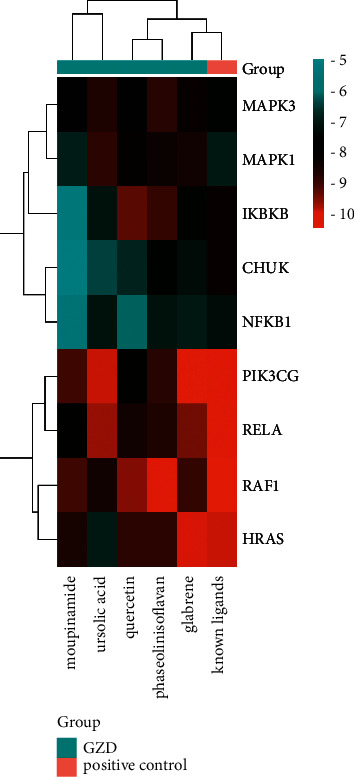
Heat map of molecular docking. The blue group represented the key five components of GZD obtained in Section 3.5, and the pink represented the positive control group (the known ligands for the top nine targets). The greener the area, the lower the binding energy and the more stable the docking result.

**Figure 10:**
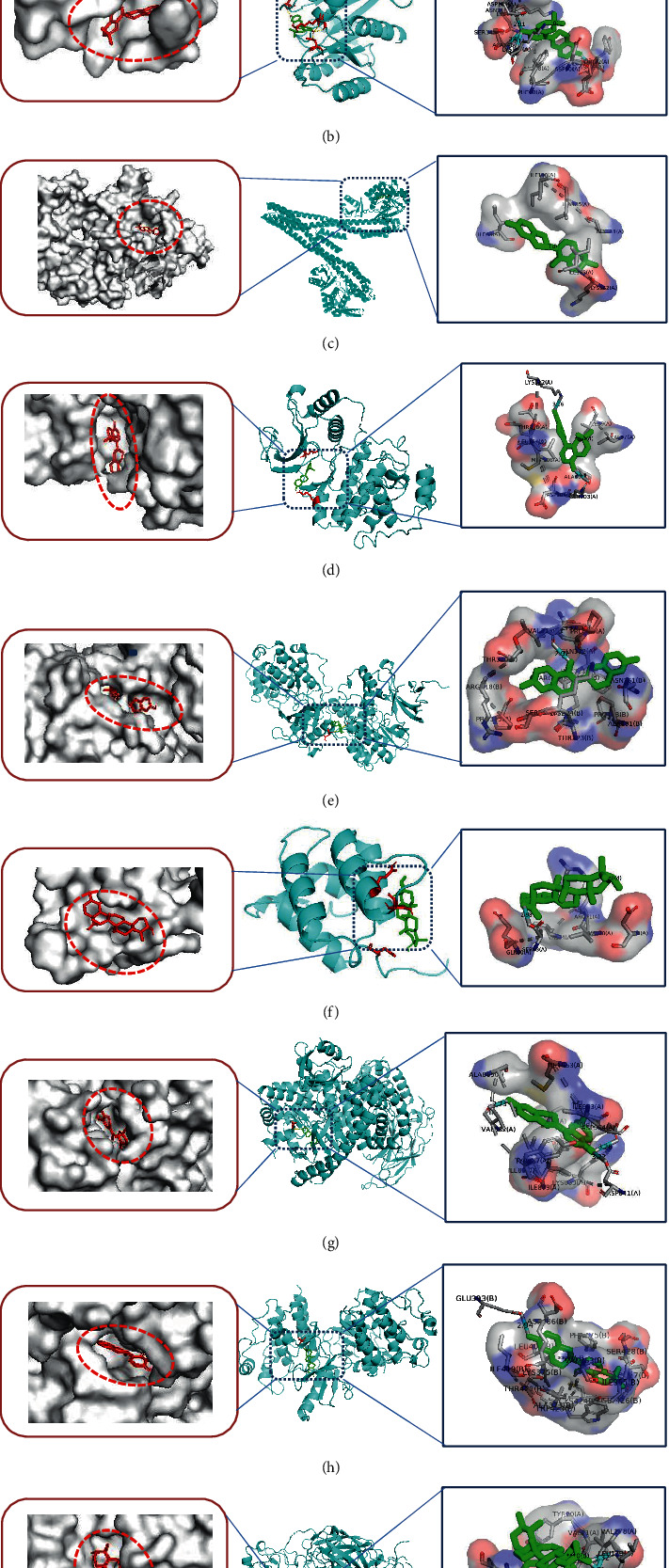
Analyses of representative target protein–ingredient docking simulation. (a) Quercetin acts on IKBKB. (b) Glabrene acts on HRAS. (c) Phaseolinisoflavan acts on CHUK. (d) Glabrene acts on MAPK1. (e) Phaseolinisoflavan acts on MAPK3. (f) Ursolic acid acts on NFKB1. (g) Glabrene acts on PIK3CG. (h) Phaseolinisoflavan acts on RAF1. (i) Ursolic acid acts on RELA. The red box on the left indicated the macromolecule binding pocket. The green in the middle picture represented the ligand, and the red represented the amino acid residues connected with hydrogen bonds. The blue box on the right indicated the binding bond of docking, and the cyan rod shape represented the hydrogen bond.

We selected the top 5 ingredients of the ingredient-target-pathway network with higher degree values; ursolic acid, quercetin, phaseolinisoflavan, moupinamide, and glabrene were regarded as small molecule ligands; for molecular docking with the top 9 targets with high degree values, MAPK1, MAPK3, PIK3CG, RAF1, HRAS, NFKB1, RELA, IKBKB, and CHUK were regarded as protein receptors, and the PDBID of genes is listed in Table 2. We used the top nine target proteins to dock with its known ligand as the longitudinal positive control. Detailed information on their known ligands was referred to the Supplementary Material Table S5 (details of the known ligand of the top targets). The docking of the positive drug (methylprednisolone) and its known target NR3C1 was shown as the horizontal positive control, where binding energy was −7.8 kcal/mol, and we proved the reliability of our method. The results of different affinity energy were shown in the figure and table (Figure 9, Table 3). We found that the affinity energy of all small molecules docked with proteins is less than or equal to −5.0 kcal/mol. They all had good binding power. We selected each group with the smallest affinity for visual analysis, and the results are shown in Figure 10.

### 3.7. Verification of the Effect of GZD on Asthma In Vitro

#### 3.7.1. Effect of GZD on the Viability of MH-S Cells

**Figure 11:**
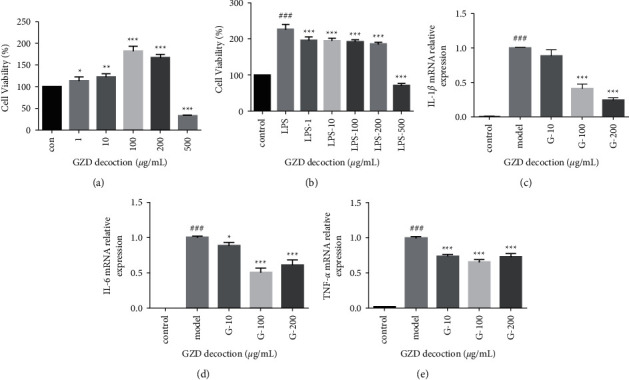
Cell viability test and the effect of GZD on the secretion of inflammatory cytokines in vitro. LPS-induced MH-S cells were employed. MH-S cells were incubated with DMSO (the same dose as GZD) for 24 hr as the control group. MH-S cells were incubated with GZD (0, 1, 10, 100, 200, and 500 *μ*g/mL) only for 24 hr as the GZD group. MH-S cells were incubated with/without LPS (5 *μ*g/mL) in the absence or presence of different concentrations with GZD (0, 1, 10, 100, 200, and 500 *μ*g/mL) for 24 hr. (a, b) The cell viability was examined using a CCK-8 assay. (c–e) The mRNA relative expressions of proinflammatory cytokines (IL-1*β*, IL-6, and TNF-*α*) in LPS-stimulated cells treated with/without GZD were detected using qPCR. ^###^*p* < 0.001 vs. the control group, ^*∗∗∗*^*p* < 0.001, ^*∗∗*^*p* < 0.01, ^*∗*^*p* < 0.05 vs. the model group.

The CCK-8 experiment results showed that the concentration range of 0–200 *μ*g/mL promoted the proliferation of MH-S cells to varying degrees, but at a concentration of 500 *μ*g/mL, the viability of MH-S cells decreased significantly. Therefore, GZD is safe and reliable within a concentration of 200 *μ*g/mL (Figure 11(a)). In addition, under the stimulation of LPS (5 *μ*g/ml), MH-S cells proliferated significantly, while the concentration range of GZD 1–200 *μ*g/mL has an inhibitory effect, but both were greater than the control group, at a concentration of 500 *μ*g/mL, the viability of MH-S cells stimulated by LPS decreased significantly, and subsequent experiments eliminated this concentration (Figure 11(b)).

#### 3.7.2. Effect of GZD on Inflammatory Factors and Key Gene mRNA Expression Levels

The MAPK cascade and NF-*κ*B pathway related to inflammation and immune regulation might play an important regulatory role in the GZD treatment of asthma [27]. Therefore, we next studied the mRNA expression of IKBKB, NAKB1, RELA (related to the NF-*κ*B pathway), MAPK1, MAPK3 (related to the MAPK pathway), RAF1, and NRAS (related to the RAS pathway) screened by network pharmacology.

**Figure 12:**
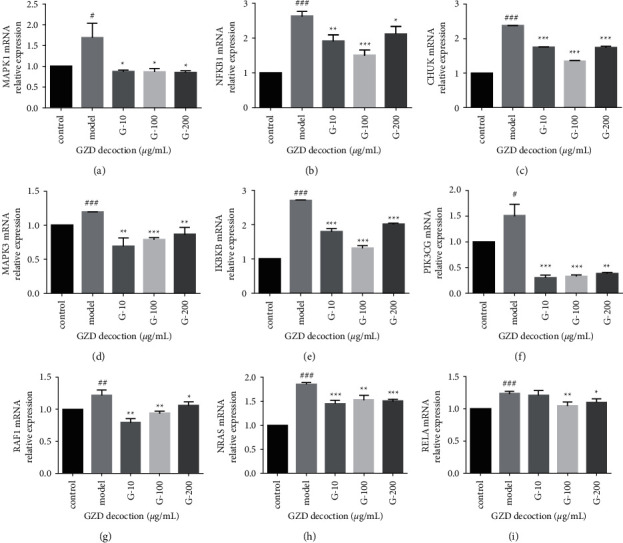
The Effect of GZD on the secretion of key genes' mRNA relative expression in vitro by using qPCR. MH-S cells were incubated with DMSO (the same dose as GZD) for 24 hr as the control group. MH-S cells were incubated with/without LPS (5 *μ*g/mL) in the absence or presence of different concentrations with GZD (0, 10, 100, 200 *μ*g/mL) for 24 hr. (a–i) mRNA relative expression of MAPK1, NFKB1, CHUK, MAPK3, IKBKB, PIK3CG, RAF1, NRAS, and RELA in different groups. ^###^*p* < 0.001, ^##^*p* < 0.01, ^#^*p* < 0.05vs. the control group, ^*∗∗∗*^*p* < 0.001, ^*∗∗*^*p* < 0.01, ^*∗*^*p* < 0.05 vs. the model group.

Compared with the control group, the secretion of inflammatory factors such as IL-1*β*, IL-6, and TNF-*α* in the cells treated with LPS was significantly upregulated, and the release of inflammatory factors was significantly reduced after administration (Figures 11(c)–11(e)). In terms of key gene MAPK1, MAPK3, NFKB1, IKBKB, NRAS, RAF1, PIK3CG, RELA, and CHUK, after administration of GZD, the increase in mRNA expression level caused by LPS stimulation was significantly reduced (Figure 12). The experimental results indicated that GZD regulated the inflammatory and immune response in asthma partly by NF-*κ*B and mitogen-activated protein kinase family signaling pathways.

## 4. Discussion

The number of patients with asthma is increasing yearly. As of now, the incidence of asthma is about 4.2% in China [4]. Traditional treatment methods have limited therapeutic effects on asthma patients and have clear side effects. Inhaled corticosteroids, the most common anti-inflammatory drugs, are currently the first choice for asthma [28, 29]. However, the huge side effects of inhaled glucocorticoids are inevitable. Long-term inhalation will inhibit the hypothalamic-pituitary-adrenal cortex axis and is prone to drug resistance. Therefore, the development of new prevention and treatment drugs for asthma needs to be solved urgently [30, 31]. The chemical components of GZD mainly include phenylpropane, monoterpenes, organic acids, flavonoids, and triterpene saponins [32]. Modern pharmacology studies have shown that GZD has two-way regulation effects on sweat glands, body temperature, immune function, gastrointestinal motility, blood pressure, etc. [13, 33, 34]. It also has anti-inflammatory, antibacterial, antiviral, antiallergic, analgesic, hypoglycemic, and cardiovascular protection effects [13, 35, 36]. It is widely used for respiratory diseases and other diseases. Feng et al. reviewed the application of GZD in colds, febrile diseases, digestive system diseases, and respiratory diseases [33]. Combined with our research, the use of GZD to treat asthma is very promising.

Asthma, featured by airway inflammation, is inseparable from inflammation and immune disorders. Jia et al. found that curcumol improved pneumonia and airway remodeling in mice with chronic asthma by inhibiting the Wnt/*β*-catenin pathway through experimental studies [37]. Lyu et al. found that *Pinellia ternata* reduced the allergic response to asthma in mice by inhibiting the activation of TH2 cells and the expression of the inflammatory factor IL-4 [38]. In the inflammatory response, proinflammatory cytokines, such as IL-1*β*, IL-6, and TNF-*α*, activate the body's immune system and promote the aggregation and activation of inflammatory cells, which plays a crucial role in the process of asthma [39]. Regulating the expression of proinflammatory cytokines is one of the main ways in which traditional Chinese medicine works. Inhibition of proinflammatory cytokines overproduction may prevent or inhibit a variety of inflammatory diseases [11, 38]. In our research, GZD could significantly reduce the release of IL-1*β*, IL-6, and TNF-*α* in vitro inflammation models. We had identified 94 core targets (Figure 5) in the PPI network through network pharmacology. Bioinformatics analysis predicted that inflammation and immunity were the main biological processes (Figure 7), and the NF-*κ*B and MAPK signaling pathways might be meaningful pathways for GZD against asthma (Figure 6). Through further analysis, we identified nine key targets including MAPK1, MAPK3, RAF1, NRAS, PIK3CG, NFKB1, IKBKB, RELA, and CHUK. Inflammation and immune-related pathways and their targets played a vital role in the GZD against asthma.

**Figure 13:**
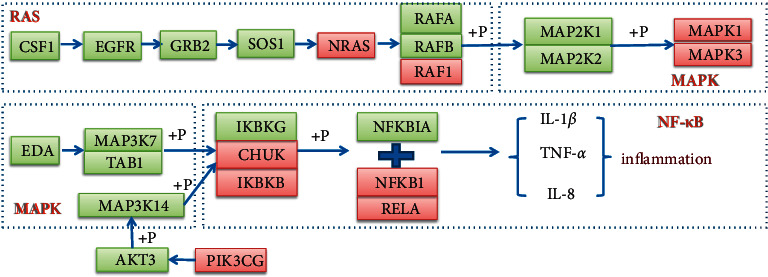
Representative pathway diagram. MAPK signaling pathway and NF-*κ*B signaling pathway were the greater representative pathways in GZD against asthma based on the results of network pharmacology. The genes in red represented important targets verified in this study. The genes in green represented other genes in these pathways.

In order to further confirm part of the possible mechanism of GZD in the treatment of asthma, we conducted a literature survey on these nine key targets. We discovered that the mitogen-activated protein kinase (MAPK) pathway included three main kinases: MAPK Kinase, MAPK Kinase Kinase, and mitogen-activated protein kinase [40]. The MAPK signaling cascade has a wide range of physiological functions including gene expression and cell death [41]. Many studies have proved that the inhibition of the MAPK signaling pathway can alleviate the development of inflammation and tumors [42–44]. The MAPK signaling pathway can be activated by a variety of proinflammatory factors [45]. Several studies reported that asthma-associated risk factors could cause pathologic activation of the MAPK pathway leading to aggravation of symptoms of asthma [46]. RAS signaling pathway as an upstream pathway of MAPK interacts with MAPK. T cell receptor signaling pathway also plays an important role in asthma; a few studies reported that allergic asthma or airway inflammation was alleviated by regulating T cell receptor signaling pathways [47, 48]. T cell receptor signaling pathway interacts with NF-*κ*B signaling pathway. The nuclear factor kappa B (NF-*κ*B) family of transcription factors is a key regulator of immune development, immune responses, inflammation, and cancer [49]. NF-*κ*B is a well-known signaling pathway that is necessary for the inflammatory response in the lung [50]. All these signal transduction pathways have crosstalk and are closely related to each other (Figure 13). In asthma, the abnormal activation of the MAPK signaling pathway and NF-*κ*B signaling pathway may play a key role in the development of the disease. Therefore, we used the key targets obtained in network pharmacology as the focus of subsequent molecular docking and in vitro verification. In the molecular docking, the binding energy of docking the positive drug (methylprednisolone) with its known target NR3C1 was −7.8 kcal/mol; this horizontal positive control showed the reliability of our method. Compared with the binding energy of proteins and their respective known ligands, our results were close to or better than the longitudinal positive control, which proved that the top five components in GZD had a stronger effect on its target and might perform a better medicinal effect, such as phaseolinisoflavan acted on MAPK3 and glabrene acted on HRAS; this showed that phaseolinisoflavan and glabrene might be the active ingredients of GZD for treating asthma.

Macrophages are important innate immune cells in the body, which can effectively swallow pathogenic microorganisms, clear senescent cells, produce various inflammatory mediators, and participate in the occurrence and development of many chronic inflammatory diseases and autoimmune diseases [51]. Mouse alveolar macrophages (MH-S) participate in important links in the development of many diseases of asthma, including the release of inflammatory mediators, the recruitment and infiltration of inflammatory cells, an abnormal antigen presentation function, abnormal phagocytosis, and clearance and oxidative stress [52]. MH-S is often used as an inflammation model in the cell for acute lung injury or chronic obstructive pulmonary disease [53, 54]. Therefore, the experimental verification part of this study chose to use the mouse alveolar macrophage cell line MH-S, which helped to better simulate the validation model.

In this experiment, LPS-stimulated MH-S cells establish an inflammation model to observe the interventional effect of GZD on LPS-induced inflammation. The results showed that the levels of IL-1*β*, IL-6, and TNF-*α* in the LPS group cells were extremely significantly increased. After treating with different concentrations of GZD, the secretion of the above-mentioned inflammatory mediators and the mRNA expression of related inflammatory genes were reduced significantly. On the one hand, GZD could inhibit the excessive secretion of inflammatory mediators caused by LPS, regulate the balance of inflammatory factors in cells, and thereby slow down the respiratory tract damage caused by inflammation. On the other hand, GZD might regulate the function of the immune system, thereby effectively alleviating asthma. To sum up, GZD might control asthma by regulating the anti-inflammatory and immunomodulatory targets in mitogen-activated protein kinase (MAPK) and nuclear factor NF-*κ*B in MH-S cells stimulated by LPS. These data laid a scientific foundation for the clinical treatment of asthma and also provided insights for the development and utilization of GZD.

## 5. Conclusions

In this study, we had initially explored the mechanism of GZD against asthma. First, we searched the relevant core targets of GZD in the treatment of asthma; then, we conducted pathway enrichment analyses and drew the “Ingredient-target-pathway” network; key compounds (ursolic acid, quercetin, phaseolinisoflavan, moupinamide, and glabrene), core targets (MAPK1, MAPK3, NFKB1, IKBKB, NRAS, RAF1, PIK3CG, RELA, and CHUK), and pathways (anti-inflammatory and immune-related) were predicted by network pharmacology. Then, molecular docking was used to predict that the components could interact with the core target proteins directly, and finally, experiments were verified that GZD could inhibit the mRNA expression of core targets. Our results showed that GZD was likely to control asthma by regulating inflammation and immune-related targets. This study provided new insights into the mechanism of GZD against asthma and gave new ideas for the clinical treatment of asthma.

## Figures and Tables

**Table 1:** Primers used in qPCR analyses of mRNA expression.

Gene	Forward	Reverse
GADPH	AAATGGTGAAGGTCGGTGTGAAC	CAACAATCTCCACTTTGCCACTG
IL-1*β*	GCAACTGTTCCTGAACTCAACT	ATCTTTTGGGGTCCGTCAACT
IL-6	AGTCCTTCCTACCCCAATTTCC	TGGTCTTGGTCCTTAGCCAC
TNF-*α*	CCCTCACACTCAGATCATCTTCT	GCTACGACGTGGGCTACAG
MAPK1	GGTTGTTCCCAAATGCTGACT	CAACTTCAATCCTCTTGTGAGGG
MAPK3	TCCGCCATGAGAATGTTATAGGC	GGTGGTGTTGATAAGCAGATTGG
RAF1	TGGACTCAAAGATGCGGTGTT	AAACCCGGATAGTATTGCTTGT
NRAS	ACTGAGTACAAACTGGTGGTGG	CGGTAAGAATCCTCTATGGTGG
PIK3CG	CGAGAGTGTCGTCACAGTGTC	TGTTCGCTTCCACAAACACAG
NFKB1	ATGGCAGACGATGATCCCTAC	TGTTGACAGTGGTATTTCTGGTG
IKBKB	CTGAAGATCGCCTGTAGCAAA	TCCATCTGTAACCAGCTCCAG
RELA	AGGCTTCTGGGCCTTATGTG	TGCTTCTCTCGCCAGGAATAC
CHUK	GTCAGGACCGTGTTCTCAAGG	GCTTCTTTGATGTTACTGAGGGC

**Table 2:** Key genes' PDBID.

Gene	PDBID
MAPK1	4G6N
MAPK3	2ZOQ
PIK3CG	4HLE
RAF1	3OMV
HRAS	4XVR
NFKB1	2DBF
RELA	1LNK
IKBKB	4KIK
CHUK	5TQW
NR3C1	3MNO

**Table 3:** Affinity energy of key components to the top genes.

Group	GZD	GZD	GZD	GZD	GZD	Longitudinal positive control
Sample	Ursolic acid	Quercetin	Phaseolinisoflavan	Moupinamide	Glabrene	The known ligands for each of top nine target proteins
MAPK1	−8.9	−8.1	−8.3	−6.9	−8.5	−7.0
MAPK3	−8.7	−7.8	−8.8	−7.9	−8.2	−7.6
PIK3CG	−9.9	−8.0	−8.8	−9.1	−10.4	−10.1
RAF1	−8.5	−9.6	−10.1	−9.1	−9.0	−10.5
HRAS	−7.0	−8.9	−8.9	−8.6	−10.0	−9.9
NFKB1	−7.1	−6.1	−7.1	−5.4	−7.0	−7.3
RELA	−9.7	−8.5	−8.7	−8.0	−9.5	−10.3
IKBKB	−7.1	−9.3	−9.0	−5.2	−7.6	−8.2
CHUK	−6.4	−6.8	−7.6	−5.0	−7.3	−8.2

## Data Availability

The datasets in this study can be obtained from the corresponding author upon reasonable request. The authors are responsible for providing the final supplementary material files that will be published along with the article.

## Supplementary Materials

Supplementary Materials Supplementary Material Table S1: 134 active compounds from TCMSP database and literature in Guizhi Decoction. Supplementary Material Table S2: drug targets information of different ingredients in Guizhi Decoction. Supplementary Material Table S3: target information at the intersection of drug targets and disease targets. Supplementary Material Table S4: core gene information filtered according to the “betweenness,” “closeness,” and “degree” values. Supplementary Material Table S5: details of the known ligand of the top targets.
