# Supplementary material for: To Predict Anti-Inflammatory and Immunomodulatory Targets of Guizhi Decoction in Treating Asthma Based on Network Pharmacology, Molecular Docking, and Experimental Validation
Source: Evid Based Complement Alternat Med. 2021 Dec 20;2021:9033842. doi: 10.1155/2021/9033842 (PMC8712140; doi:10.1155/2021/9033842)
Supplement: Supplementary Materials — Supplementary Material Table S1: 134 active compounds from TCMSP database and literature in Guizhi Decoction. Supplementary Material Table S2: drug targets information of different ingredients in Guizhi Decoction. Supplementary Material Table S3: target information at the intersection of drug targets and disease targets. Supplementary Material Table S4: core gene information filtered according to the “betweenness,” “closeness,” and “degree” values. Supplementary Material Table S5: details of the known ligand of the top targets. [file 9033842.f1.zip › 9033842.f1/Supplementary Material Table S2 (1).docx]

***Supplementary Material***

**Table S2** Drug targets information of different ingredients in Guizhi Decoction

| **Gene** | **Herb** | **Compound** | **source** |
| --- | --- | --- | --- |
| TRPA1 | guizhi | Cinnamaldehyde | Swisstarget prediction |
| HCAR2 | guizhi | Cinnamaldehyde | Swisstarget prediction |
| F3 | guizhi | Cinnamaldehyde | Swisstarget prediction |
| ADH1B | guizhi | Cinnamaldehyde | Swisstarget prediction |
| ADH1C | guizhi | Cinnamaldehyde | Swisstarget prediction |
| CYP2A6 | guizhi | Cinnamaldehyde | Swisstarget prediction |
| RELA | guizhi | Cinnamaldehyde | Swisstarget prediction |
| ADH1A | guizhi | Cinnamaldehyde | Swisstarget prediction |
| TLR4 | guizhi | Cinnamaldehyde | Swisstarget prediction |
| HDAC6 | guizhi | Cinnamaldehyde | Swisstarget prediction |
| HDAC2 | guizhi | Cinnamaldehyde | Swisstarget prediction |
| CA2 | guizhi | Cinnamaldehyde | Swisstarget prediction |
| CA1 | guizhi | Cinnamaldehyde | Swisstarget prediction |
| AKR1B1 | guizhi | Cinnamic acid | Swisstarget prediction |
| ESR2 | guizhi | Cinnamic acid | Swisstarget prediction |
| CA6 | guizhi | Cinnamic acid | Swisstarget prediction |
| SLC16A1 | guizhi | Cinnamic acid | Swisstarget prediction |
| CA7 | guizhi | Cinnamic acid | Swisstarget prediction |
| CA12 | guizhi | Cinnamic acid | Swisstarget prediction |
| CA14 | guizhi | Cinnamic acid | Swisstarget prediction |
| CA9 | guizhi | Cinnamic acid | Swisstarget prediction |
| CA4 | guizhi | Cinnamic acid | Swisstarget prediction |
| CA5B | guizhi | Cinnamic acid | Swisstarget prediction |
| CA5A | guizhi | Cinnamic acid | Swisstarget prediction |
| CA3 | guizhi | Cinnamic acid | Swisstarget prediction |
| ALOX5 | guizhi | Cinnamic acid | Swisstarget prediction |
| MMP9 | guizhi | Cinnamic acid | Swisstarget prediction |
| MMP1 | guizhi | Cinnamic acid | Swisstarget prediction |
| MMP2 | guizhi | Cinnamic acid | Swisstarget prediction |
| PTPN1 | guizhi | Cinnamic acid | Swisstarget prediction |
| AKR1C1 | guizhi | Cinnamic acid | Swisstarget prediction |
| AKR1B10 | guizhi | Cinnamic acid | Swisstarget prediction |
| CAPN2 | guizhi | Cinnamic acid | Swisstarget prediction |
| CAPN1 | guizhi | Cinnamic acid | Swisstarget prediction |
| IDO1 | guizhi | Cinnamic acid | Swisstarget prediction |
| DAO | guizhi | Cinnamic acid | Swisstarget prediction |
| RNPEP | guizhi | Cinnamic acid | Swisstarget prediction |
| EGFR | guizhi | Cinnamic acid | Swisstarget prediction |
| CYP19A1 | guizhi | Tetradecanal | Swisstarget prediction |
| CES1 | guizhi | Tetradecanal | Swisstarget prediction |
| CES2 | guizhi | Tetradecanal | Swisstarget prediction |
| NR1I3 | guizhi | Tetradecanal | Swisstarget prediction |
| AR | guizhi | Tetradecanal | Swisstarget prediction |
| EPHX1 | guizhi | Tetradecanal | Swisstarget prediction |
| PGR | guizhi | Tetradecanal | Swisstarget prediction |
| SRD5A2 | guizhi | Tetradecanal | Swisstarget prediction |
| SRD5A1 | guizhi | Tetradecanal | Swisstarget prediction |
| PAOX | guizhi | Tetradecanal | Swisstarget prediction |
| ICMT | guizhi | Tetradecanal | Swisstarget prediction |
| TBXAS1 | guizhi | Tetradecanal | Swisstarget prediction |
| ALDH2 | guizhi | Tetradecanal | Swisstarget prediction |
| ACACB | guizhi | Tetradecanal | Swisstarget prediction |
| RORC | guizhi | Tetradecanal | Swisstarget prediction |
| SCN9A | guizhi | Tetradecanal | Swisstarget prediction |
| MTNR1A | guizhi | Tetradecanal | Swisstarget prediction |
| MTNR1B | guizhi | Tetradecanal | Swisstarget prediction |
| KDR | guizhi | Tetradecanal | Swisstarget prediction |
| MCHR1 | guizhi | Tetradecanal | Swisstarget prediction |
| HSD3B1 | guizhi | Tetradecanal | Swisstarget prediction |
| SHBG | shaoyao | (3S,5R,8R,9R,10S,14S)-3,17-dihydroxy-4,4,8,10,14-pentamethyl-2,3,5,6,7,9-hexahydro-1H-cyclopenta[a]phenanthrene-15,16-dione | Swisstarget prediction |
| GABBR1 | shaoyao | (3S,5R,8R,9R,10S,14S)-3,17-dihydroxy-4,4,8,10,14-pentamethyl-2,3,5,6,7,9-hexahydro-1H-cyclopenta[a]phenanthrene-15,16-dione | Swisstarget prediction |
| CYP2D6 | shaoyao | (3S,5R,8R,9R,10S,14S)-3,17-dihydroxy-4,4,8,10,14-pentamethyl-2,3,5,6,7,9-hexahydro-1H-cyclopenta[a]phenanthrene-15,16-dione | Swisstarget prediction |
| CYP2C9 | shaoyao | (3S,5R,8R,9R,10S,14S)-3,17-dihydroxy-4,4,8,10,14-pentamethyl-2,3,5,6,7,9-hexahydro-1H-cyclopenta[a]phenanthrene-15,16-dione | Swisstarget prediction |
| NR3C1 | shaoyao | (3S,5R,8R,9R,10S,14S)-3,17-dihydroxy-4,4,8,10,14-pentamethyl-2,3,5,6,7,9-hexahydro-1H-cyclopenta[a]phenanthrene-15,16-dione | Swisstarget prediction |
| ESRRA | shaoyao | (3S,5R,8R,9R,10S,14S)-3,17-dihydroxy-4,4,8,10,14-pentamethyl-2,3,5,6,7,9-hexahydro-1H-cyclopenta[a]phenanthrene-15,16-dione | Swisstarget prediction |
| ESRRB | shaoyao | (3S,5R,8R,9R,10S,14S)-3,17-dihydroxy-4,4,8,10,14-pentamethyl-2,3,5,6,7,9-hexahydro-1H-cyclopenta[a]phenanthrene-15,16-dione | Swisstarget prediction |
| GPER1 | shaoyao | (3S,5R,8R,9R,10S,14S)-3,17-dihydroxy-4,4,8,10,14-pentamethyl-2,3,5,6,7,9-hexahydro-1H-cyclopenta[a]phenanthrene-15,16-dione | Swisstarget prediction |
| ADCY10 | shaoyao | (3S,5R,8R,9R,10S,14S)-3,17-dihydroxy-4,4,8,10,14-pentamethyl-2,3,5,6,7,9-hexahydro-1H-cyclopenta[a]phenanthrene-15,16-dione | Swisstarget prediction |
| HAO1 | shaoyao | (3S,5R,8R,9R,10S,14S)-3,17-dihydroxy-4,4,8,10,14-pentamethyl-2,3,5,6,7,9-hexahydro-1H-cyclopenta[a]phenanthrene-15,16-dione | Swisstarget prediction |
| ESR1 | shaoyao | (3S,5R,8R,9R,10S,14S)-3,17-dihydroxy-4,4,8,10,14-pentamethyl-2,3,5,6,7,9-hexahydro-1H-cyclopenta[a]phenanthrene-15,16-dione | Swisstarget prediction |
| CHRM1 | shaoyao | (3S,5R,8R,9R,10S,14S)-3,17-dihydroxy-4,4,8,10,14-pentamethyl-2,3,5,6,7,9-hexahydro-1H-cyclopenta[a]phenanthrene-15,16-dione | Swisstarget prediction |
| LGALS3 | shaoyao | Lactiflorin | Swisstarget prediction |
| LGALS9 | shaoyao | Lactiflorin | Swisstarget prediction |
| PTAFR | shaoyao | Lactiflorin | Swisstarget prediction |
| ADORA3 | shaoyao | Lactiflorin | Swisstarget prediction |
| HSPA8 | shaoyao | Lactiflorin | Swisstarget prediction |
| HSPA5 | shaoyao | Lactiflorin | Swisstarget prediction |
| ADK | shaoyao | Lactiflorin | Swisstarget prediction |
| PPM1A | shaoyao | Lactiflorin | Swisstarget prediction |
| MME | shaoyao | Lactiflorin | Swisstarget prediction |
| ECE1 | shaoyao | Lactiflorin | Swisstarget prediction |
| ADORA2A | shaoyao | Lactiflorin | Swisstarget prediction |
| SLC29A1 | shaoyao | Lactiflorin | Swisstarget prediction |
| SLC6A3 | shaoyao | Lactiflorin | Swisstarget prediction |
| LGALS7 | shaoyao | Lactiflorin | Swisstarget prediction |
| SLC28A2 | shaoyao | Lactiflorin | Swisstarget prediction |
| VEGFA | shaoyao | Lactiflorin | Swisstarget prediction |
| FGF1 | shaoyao | Lactiflorin | Swisstarget prediction |
| FGF2 | shaoyao | Lactiflorin | Swisstarget prediction |
| HPSE | shaoyao | Lactiflorin | Swisstarget prediction |
| HK2 | shaoyao | Lactiflorin | Swisstarget prediction |
| HK1 | shaoyao | Lactiflorin | Swisstarget prediction |
| HSP90AA1 | shaoyao | Lactiflorin | Swisstarget prediction |
| PFKFB3 | shaoyao | Lactiflorin | Swisstarget prediction |
| GBA | shaoyao | Lactiflorin | Swisstarget prediction |
| ABCB1 | shaoyao | Lactiflorin | Swisstarget prediction |
| SLC5A11 | shaoyao | Lactiflorin | Swisstarget prediction |
| ADORA2B | shaoyao | Lactiflorin | Swisstarget prediction |
| GAPDH | shaoyao | Lactiflorin | Swisstarget prediction |
| MAP2K1 | shaoyao | Lactiflorin | Swisstarget prediction |
| GLRA1 | shaoyao | Lactiflorin | Swisstarget prediction |
| GLRA2 | shaoyao | Lactiflorin | Swisstarget prediction |
| SSTR5 | shaoyao | Lactiflorin | Swisstarget prediction |
| SSTR2 | shaoyao | Lactiflorin | Swisstarget prediction |
| SSTR4 | shaoyao | Lactiflorin | Swisstarget prediction |
| SSTR1 | shaoyao | Lactiflorin | Swisstarget prediction |
| SSTR3 | shaoyao | Lactiflorin | Swisstarget prediction |
| IRAK4 | shaoyao | Lactiflorin | Swisstarget prediction |
| FDFT1 | shaoyao | Lactiflorin | Swisstarget prediction |
| SLC6A2 | shaoyao | paeoniflorin | Swisstarget prediction |
| F10 | shaoyao | paeoniflorin | Swisstarget prediction |
| AMY1A | shaoyao | paeoniflorin | Swisstarget prediction |
| SERPINE1 | shaoyao | paeoniflorin | Swisstarget prediction |
| PTPN2 | shaoyao | paeoniflorin | Swisstarget prediction |
| BACE1 | shaoyao | paeoniflorin | Swisstarget prediction |
| SQLE | shaoyao | paeoniflorin | Swisstarget prediction |
| SELP | shaoyao | paeoniflorin | Swisstarget prediction |
| PRKCA | shaoyao | albiflorin_qt | Swisstarget prediction |
| MMP13 | shaoyao | albiflorin_qt | Swisstarget prediction |
| MMP7 | shaoyao | albiflorin_qt | Swisstarget prediction |
| MMP8 | shaoyao | albiflorin_qt | Swisstarget prediction |
| TOP1 | shaoyao | albiflorin_qt | Swisstarget prediction |
| NRAS | shaoyao | albiflorin_qt | Swisstarget prediction |
| AGTR1 | shaoyao | albiflorin_qt | Swisstarget prediction |
| IMPDH1 | shaoyao | albiflorin_qt | Swisstarget prediction |
| HRAS | shaoyao | albiflorin_qt | Swisstarget prediction |
| PRKCG | shaoyao | albiflorin_qt | Swisstarget prediction |
| PRKCH | shaoyao | albiflorin_qt | Swisstarget prediction |
| IGFBP3 | shaoyao | albiflorin_qt | Swisstarget prediction |
| OGA | shaoyao | albiflorin_qt | Swisstarget prediction |
| MGMT | shaoyao | albiflorin_qt | Swisstarget prediction |
| PARP1 | shaoyao | albiflorin_qt | Swisstarget prediction |
| MMP3 | shaoyao | albiflorin_qt | Swisstarget prediction |
| ADAM17 | shaoyao | albiflorin_qt | Swisstarget prediction |
| ABL1 | shaoyao | albiflorin_qt | Swisstarget prediction |
| EPHA2 | shaoyao | albiflorin_qt | Swisstarget prediction |
| LCK | shaoyao | albiflorin_qt | Swisstarget prediction |
| SRC | shaoyao | albiflorin_qt | Swisstarget prediction |
| MAP3K9 | shaoyao | albiflorin_qt | Swisstarget prediction |
| FGFR1 | shaoyao | albiflorin_qt | Swisstarget prediction |
| AURKA | shaoyao | albiflorin_qt | Swisstarget prediction |
| BTK | shaoyao | albiflorin_qt | Swisstarget prediction |
| NADK | shaoyao | albiflorin_qt | Swisstarget prediction |
| FUCA1 | shaoyao | albiflorin_qt | Swisstarget prediction |
| SLC28A3 | shaoyao | albiflorin_qt | Swisstarget prediction |
| AMD1 | shaoyao | albiflorin_qt | Swisstarget prediction |
| UBA2 | shaoyao | Mairin | Swisstarget prediction |
| SAE1 | shaoyao | Mairin | Swisstarget prediction |
| POLB | shaoyao | Mairin | Swisstarget prediction |
| HSD11B1 | shaoyao | Mairin | Swisstarget prediction |
| PTGES | shaoyao | Mairin | Swisstarget prediction |
| NR1H4 | shaoyao | Mairin | Swisstarget prediction |
| CDC25C | shaoyao | Mairin | Swisstarget prediction |
| GPBAR1 | shaoyao | Mairin | Swisstarget prediction |
| TOP2A | shaoyao | Mairin | Swisstarget prediction |
| CDC25A | shaoyao | Mairin | Swisstarget prediction |
| TERT | shaoyao | Mairin | Swisstarget prediction |
| PTPRF | shaoyao | Mairin | Swisstarget prediction |
| ACP1 | shaoyao | Mairin | Swisstarget prediction |
| FABP4 | shaoyao | Mairin | Swisstarget prediction |
| FAAH | shaoyao | Mairin | Swisstarget prediction |
| FABP3 | shaoyao | Mairin | Swisstarget prediction |
| FABP5 | shaoyao | Mairin | Swisstarget prediction |
| PPARD | shaoyao | Mairin | Swisstarget prediction |
| FABP1 | shaoyao | Mairin | Swisstarget prediction |
| PTPN11 | shaoyao | Mairin | Swisstarget prediction |
| UGT2B7 | shaoyao | Mairin | Swisstarget prediction |
| SCD | shaoyao | Mairin | Swisstarget prediction |
| SERPINA6 | shaoyao | Mairin | Swisstarget prediction |
| CYP51A1 | shaoyao | Mairin | Swisstarget prediction |
| HSD17B3 | shaoyao | Mairin | Swisstarget prediction |
| PLA2G1B | shaoyao | Mairin | Swisstarget prediction |
| CDC25B | shaoyao | Mairin | Swisstarget prediction |
| PDE4D | shaoyao | Mairin | Swisstarget prediction |
| NPC1L1 | shaoyao | Mairin | Swisstarget prediction |
| SIGMAR1 | shaoyao | Mairin | Swisstarget prediction |
| CYP17A1 | shaoyao | Mairin | Swisstarget prediction |
| VDR | shaoyao | Mairin | Swisstarget prediction |
| GABRG2 | shaoyao | Mairin | Swisstarget prediction |
| GABRB2 | shaoyao | Mairin | Swisstarget prediction |
| GABRA2 | shaoyao | Mairin | Swisstarget prediction |
| PPARG | shaoyao | Mairin | Swisstarget prediction |
| FFAR1 | shaoyao | Mairin | Swisstarget prediction |
| FABP2 | shaoyao | Mairin | Swisstarget prediction |
| HSF1 | shaoyao | Mairin | Swisstarget prediction |
| ACHE | shaoyao | kaempferol | Swisstarget prediction |
| ADORA1 | shaoyao | kaempferol | Swisstarget prediction |
| AKR1A1 | shaoyao | kaempferol | Swisstarget prediction |
| AKR1C2 | shaoyao | kaempferol | Swisstarget prediction |
| AKR1C4 | shaoyao | kaempferol | Swisstarget prediction |
| AKR1C3 | shaoyao | kaempferol | Swisstarget prediction |
| ALK | shaoyao | kaempferol | Swisstarget prediction |
| ALOX12 | shaoyao | kaempferol | Swisstarget prediction |
| ALOX15 | shaoyao | kaempferol | Swisstarget prediction |
| ARG1 | shaoyao | kaempferol | Swisstarget prediction |
| AHR | shaoyao | kaempferol | Swisstarget prediction |
| ABCG2 | shaoyao | kaempferol | Swisstarget prediction |
| APP | shaoyao | kaempferol | Swisstarget prediction |
| BCHE | shaoyao | kaempferol | Swisstarget prediction |
| CAMK2B | shaoyao | kaempferol | Swisstarget prediction |
| CA13 | shaoyao | kaempferol | Swisstarget prediction |
| CSNK2A1 | shaoyao | kaempferol | Swisstarget prediction |
| CDK1 | shaoyao | kaempferol | Swisstarget prediction |
| CCNB1 | shaoyao | kaempferol | Swisstarget prediction |
| CCNB2 | shaoyao | kaempferol | Swisstarget prediction |
| CCNB3 | shaoyao | kaempferol | Swisstarget prediction |
| CDK2 | shaoyao | kaempferol | Swisstarget prediction |
| CDK5 | shaoyao | kaempferol | Swisstarget prediction |
| CDK5R1 | shaoyao | kaempferol | Swisstarget prediction |
| CDK6 | shaoyao | kaempferol | Swisstarget prediction |
| PTGS2 | shaoyao | kaempferol | Swisstarget prediction |
| CFTR | shaoyao | kaempferol | Swisstarget prediction |
| CYP1B1 | shaoyao | kaempferol | Swisstarget prediction |
| DAPK1 | shaoyao | kaempferol | Swisstarget prediction |
| MPG | shaoyao | kaempferol | Swisstarget prediction |
| DRD4 | shaoyao | kaempferol | Swisstarget prediction |
| HSD17B1 | shaoyao | kaempferol | Swisstarget prediction |
| HSD17B2 | shaoyao | kaempferol | Swisstarget prediction |
| PTK2 | shaoyao | kaempferol | Swisstarget prediction |
| GRK6 | shaoyao | kaempferol | Swisstarget prediction |
| GPR35 | shaoyao | kaempferol | Swisstarget prediction |
| GSK3B | shaoyao | kaempferol | Swisstarget prediction |
| GLO1 | shaoyao | kaempferol | Swisstarget prediction |
| MET | shaoyao | kaempferol | Swisstarget prediction |
| IGF1R | shaoyao | kaempferol | Swisstarget prediction |
| CXCR1 | shaoyao | kaempferol | Swisstarget prediction |
| PYGL | shaoyao | kaempferol | Swisstarget prediction |
| CD38 | shaoyao | kaempferol | Swisstarget prediction |
| MMP12 | shaoyao | kaempferol | Swisstarget prediction |
| MAOA | shaoyao | kaempferol | Swisstarget prediction |
| ABCC1 | shaoyao | kaempferol | Swisstarget prediction |
| MPO | shaoyao | kaempferol | Swisstarget prediction |
| NOX4 | shaoyao | kaempferol | Swisstarget prediction |
| NUAK1 | shaoyao | kaempferol | Swisstarget prediction |
| PIK3R1 | shaoyao | kaempferol | Swisstarget prediction |
| PKN1 | shaoyao | kaempferol | Swisstarget prediction |
| PTPRS | shaoyao | kaempferol | Swisstarget prediction |
| AKT1 | shaoyao | kaempferol | Swisstarget prediction |
| AURKB | shaoyao | kaempferol | Swisstarget prediction |
| NEK2 | shaoyao | kaempferol | Swisstarget prediction |
| NEK6 | shaoyao | kaempferol | Swisstarget prediction |
| PIM1 | shaoyao | kaempferol | Swisstarget prediction |
| PLK1 | shaoyao | kaempferol | Swisstarget prediction |
| SLC22A12 | shaoyao | kaempferol | Swisstarget prediction |
| TNKS | shaoyao | kaempferol | Swisstarget prediction |
| TNKS2 | shaoyao | kaempferol | Swisstarget prediction |
| F2 | shaoyao | kaempferol | Swisstarget prediction |
| TTR | shaoyao | kaempferol | Swisstarget prediction |
| TYR | shaoyao | kaempferol | Swisstarget prediction |
| FLT3 | shaoyao | kaempferol | Swisstarget prediction |
| AXL | shaoyao | kaempferol | Swisstarget prediction |
| SYK | shaoyao | kaempferol | Swisstarget prediction |
| AVPR2 | shaoyao | kaempferol | Swisstarget prediction |
| XDH | shaoyao | kaempferol | Swisstarget prediction |
| ERN1 | shaoyao | Paeonol | Swisstarget prediction |
| HMGCR | shaoyao | Paeonol | Swisstarget prediction |
| EP300 | shaoyao | Paeonol | Swisstarget prediction |
| ALPG | shaoyao | Paeonol | Swisstarget prediction |
| PLAA | shaoyao | Paeonol | Swisstarget prediction |
| MAOB | shaoyao | Paeonol | Swisstarget prediction |
| FASN | shaoyao | Paeonol | Swisstarget prediction |
| KCNMA1 | shaoyao | Paeonol | Swisstarget prediction |
| CISD1 | shaoyao | Paeonol | Swisstarget prediction |
| PIM3 | shaoyao | Paeonol | Swisstarget prediction |
| MB | shaoyao | Paeonol | Swisstarget prediction |
| NAT1 | shaoyao | Paeonol | Swisstarget prediction |
| ASF1A | shaoyao | Paeonol | Swisstarget prediction |
| PLEC | shaoyao | Paeonol | Swisstarget prediction |
| CSNK1A1 | shaoyao | Paeonol | Swisstarget prediction |
| CSNK1D | shaoyao | Paeonol | Swisstarget prediction |
| PTPN22 | shaoyao | Paeonol | Swisstarget prediction |
| RPS6KA3 | gancao | Inermine | Swisstarget prediction |
| EZR | gancao | Inermine | Swisstarget prediction |
| CLK1 | gancao | Inermine | Swisstarget prediction |
| DYRK1B | gancao | Inermine | Swisstarget prediction |
| RET | gancao | Inermine | Swisstarget prediction |
| TRPM8 | gancao | Inermine | Swisstarget prediction |
| CHEK1 | gancao | Inermine | Swisstarget prediction |
| WEE1 | gancao | Inermine | Swisstarget prediction |
| CHEK2 | gancao | Inermine | Swisstarget prediction |
| RPS6KA1 | gancao | Inermine | Swisstarget prediction |
| DGAT1 | gancao | Inermine | Swisstarget prediction |
| IKBKB | gancao | Inermine | Swisstarget prediction |
| MTOR | gancao | Inermine | Swisstarget prediction |
| PIK3CD | gancao | Inermine | Swisstarget prediction |
| PIK3CB | gancao | Inermine | Swisstarget prediction |
| PIK3CG | gancao | Inermine | Swisstarget prediction |
| PIK3CA | gancao | Inermine | Swisstarget prediction |
| PGF | gancao | Inermine | Swisstarget prediction |
| GSTP1 | gancao | Inermine | Swisstarget prediction |
| GSTM2 | gancao | Inermine | Swisstarget prediction |
| TBK1 | gancao | Inermine | Swisstarget prediction |
| LNPEP | gancao | Inermine | Swisstarget prediction |
| TUBB1 | gancao | Inermine | Swisstarget prediction |
| ADCY5 | gancao | Inermine | Swisstarget prediction |
| MAP3K8 | gancao | Inermine | Swisstarget prediction |
| CCND1 | gancao | Inermine | Swisstarget prediction |
| CDK4 | gancao | Inermine | Swisstarget prediction |
| CCND2 | gancao | Inermine | Swisstarget prediction |
| CCND3 | gancao | Inermine | Swisstarget prediction |
| PIM2 | gancao | Inermine | Swisstarget prediction |
| RAF1 | gancao | Inermine | Swisstarget prediction |
| MIF | gancao | Inermine | Swisstarget prediction |
| JAK3 | gancao | Inermine | Swisstarget prediction |
| BRAF | gancao | Inermine | Swisstarget prediction |
| MAPKAPK2 | gancao | Inermine | Swisstarget prediction |
| HDAC4 | gancao | Inermine | Swisstarget prediction |
| GRK2 | gancao | Inermine | Swisstarget prediction |
| IMPDH2 | gancao | Inermine | Swisstarget prediction |
| NEK1 | gancao | Inermine | Swisstarget prediction |
| CBR1 | gancao | DFV | Swisstarget prediction |
| TAS2R31 | gancao | DFV | Swisstarget prediction |
| PTGS1 | gancao | DFV | Swisstarget prediction |
| SLC5A2 | gancao | DFV | Swisstarget prediction |
| PLA2G5 | gancao | DFV | Swisstarget prediction |
| PLA2G10 | gancao | DFV | Swisstarget prediction |
| GRM5 | gancao | DFV | Swisstarget prediction |
| RXRA | gancao | DFV | Swisstarget prediction |
| KLK1 | gancao | DFV | Swisstarget prediction |
| KLK2 | gancao | DFV | Swisstarget prediction |
| EDNRA | gancao | DFV | Swisstarget prediction |
| HSD17B14 | gancao | DFV | Swisstarget prediction |
| CHRNA7 | gancao | DFV | Swisstarget prediction |
| KIT | gancao | DFV | Swisstarget prediction |
| DYRK1A | gancao | DFV | Swisstarget prediction |
| PLA2G2A | gancao | DFV | Swisstarget prediction |
| YWHAG | gancao | DFV | Swisstarget prediction |
| PTGER1 | gancao | DFV | Swisstarget prediction |
| PTGER4 | gancao | DFV | Swisstarget prediction |
| PTGER2 | gancao | DFV | Swisstarget prediction |
| PTGER3 | gancao | DFV | Swisstarget prediction |
| BCL2L1 | gancao | DFV | Swisstarget prediction |
| VCP | gancao | DFV | Swisstarget prediction |
| RPS6KA5 | gancao | DFV | Swisstarget prediction |
| INSR | gancao | DFV | Swisstarget prediction |
| NQO2 | gancao | DFV | Swisstarget prediction |
| GRM2 | gancao | DFV | Swisstarget prediction |
| PDPK1 | gancao | DFV | Swisstarget prediction |
| DNM1 | gancao | DFV | Swisstarget prediction |
| CYP3A4 | gancao | DFV | Swisstarget prediction |
| DUSP3 | gancao | DFV | Swisstarget prediction |
| HNF4A | gancao | DFV | Swisstarget prediction |
| GCGR | gancao | DFV | Swisstarget prediction |
| CDK3 | gancao | DFV | Swisstarget prediction |
| CCNE1 | gancao | DFV | Swisstarget prediction |
| CALM1 | gancao | DFV | Swisstarget prediction |
| HDAC5 | gancao | DFV | Swisstarget prediction |
| HDAC7 | gancao | DFV | Swisstarget prediction |
| HDAC9 | gancao | DFV | Swisstarget prediction |
| XPO1 | gancao | Glycyrol | Swisstarget prediction |
| HSP90B1 | gancao | Glycyrol | Swisstarget prediction |
| HSP90AB1 | gancao | Glycyrol | Swisstarget prediction |
| FNTA FNTB | gancao | Glycyrol | Swisstarget prediction |
| CCR4 | gancao | Glycyrol | Swisstarget prediction |
| NFKB1 | gancao | Glycyrol | Swisstarget prediction |
| EGLN1 | gancao | Glycyrol | Swisstarget prediction |
| NOS2 | gancao | Glycyrol | Swisstarget prediction |
| ROCK2 | gancao | Glycyrol | Swisstarget prediction |
| PDE10A | gancao | Glycyrol | Swisstarget prediction |
| GPR84 | gancao | Glycyrol | Swisstarget prediction |
| MPI | gancao | Glycyrol | Swisstarget prediction |
| IDH1 | gancao | Glycyrol | Swisstarget prediction |
| CXCR2 | gancao | Glycyrol | Swisstarget prediction |
| PLAU | gancao | Glycyrol | Swisstarget prediction |
| MCL1 | gancao | Jaranol | Swisstarget prediction |
| PLG | gancao | Jaranol | Swisstarget prediction |
| OPRD1 | gancao | Jaranol | Swisstarget prediction |
| OPRM1 | gancao | Jaranol | Swisstarget prediction |
| KDM4E | gancao | Jaranol | Swisstarget prediction |
| APEX1 | gancao | Jaranol | Swisstarget prediction |
| MYLK | gancao | Jaranol | Swisstarget prediction |
| ODC1 | gancao | Jaranol | Swisstarget prediction |
| NAE1 | gancao | Jaranol | Swisstarget prediction |
| HTT | gancao | Medicarpin | Swisstarget prediction |
| MKNK1 | gancao | Medicarpin | Swisstarget prediction |
| EIF4A1 | gancao | Medicarpin | Swisstarget prediction |
| COMT | gancao | Medicarpin | Swisstarget prediction |
| CASP3 | gancao | Medicarpin | Swisstarget prediction |
| CASP7 | gancao | Medicarpin | Swisstarget prediction |
| TYMS | gancao | Medicarpin | Swisstarget prediction |
| PDE4B | gancao | Medicarpin | Swisstarget prediction |
| CYP11B1 | gancao | Medicarpin | Swisstarget prediction |
| PDE4A | gancao | Medicarpin | Swisstarget prediction |
| CYP11B2 | gancao | Medicarpin | Swisstarget prediction |
| PDE4C | gancao | Medicarpin | Swisstarget prediction |
| ALPL | gancao | Medicarpin | Swisstarget prediction |
| PRKDC | gancao | Medicarpin | Swisstarget prediction |
| HCK | gancao | Medicarpin | Swisstarget prediction |
| RPS6KA2 | gancao | Medicarpin | Swisstarget prediction |
| KDM4C | gancao | Medicarpin | Swisstarget prediction |
| TUBB3 | gancao | Medicarpin | Swisstarget prediction |
| EPHB2 | gancao | Medicarpin | Swisstarget prediction |
| GRIA1 | gancao | Medicarpin | Swisstarget prediction |
| GCK | gancao | Medicarpin | Swisstarget prediction |
| GABRA5 | gancao | Medicarpin | Swisstarget prediction |
| GABRB3 | gancao | Medicarpin | Swisstarget prediction |
| PIK3CA | gancao | Medicarpin | Swisstarget prediction |
| CTSS | gancao | Medicarpin | Swisstarget prediction |
| PGK1 | gancao | Medicarpin | Swisstarget prediction |
| MPEG1 | gancao | Medicarpin | Swisstarget prediction |
| XIAP | gancao | Medicarpin | Swisstarget prediction |
| MBD2 | gancao | Medicarpin | Swisstarget prediction |
| EPHA3 | gancao | Medicarpin | Swisstarget prediction |
| EPHB4 | gancao | Medicarpin | Swisstarget prediction |
| MAPT | gancao | isorhamnetin | Swisstarget prediction |
| PDE5A | gancao | Lupiwighteone | Swisstarget prediction |
| MGAM | gancao | Lupiwighteone | Swisstarget prediction |
| HTR2A | gancao | Lupiwighteone | Swisstarget prediction |
| HTR2C | gancao | Lupiwighteone | Swisstarget prediction |
| BCL2 | gancao | Lupiwighteone | Swisstarget prediction |
| IL2 | gancao | Lupiwighteone | Swisstarget prediction |
| SIRT1 | gancao | Lupiwighteone | Swisstarget prediction |
| CCNA1 | gancao | 7-Methoxy-2-methyl isoflavone | Swisstarget prediction |
| CCNA2 | gancao | 7-Methoxy-2-methyl isoflavone | Swisstarget prediction |
| CDK2 | gancao | 7-Methoxy-2-methyl isoflavone | Swisstarget prediction |
| DRD1 | gancao | 7-Methoxy-2-methyl isoflavone | Swisstarget prediction |
| SLC1A3 | gancao | 7-Methoxy-2-methyl isoflavone | Swisstarget prediction |
| BCAT2 | gancao | 7-Methoxy-2-methyl isoflavone | Swisstarget prediction |
| PNMT | gancao | 7-Methoxy-2-methyl isoflavone | Swisstarget prediction |
| GRK3 | gancao | 7-Methoxy-2-methyl isoflavone | Swisstarget prediction |
| HPGD | gancao | 7-Methoxy-2-methyl isoflavone | Swisstarget prediction |
| GRK5 | gancao | 7-Methoxy-2-methyl isoflavone | Swisstarget prediction |
| CHRNB2 | gancao | 7-Methoxy-2-methyl isoflavone | Swisstarget prediction |
| CHRNA4 | gancao | 7-Methoxy-2-methyl isoflavone | Swisstarget prediction |
| CCNE1 | gancao | 7-Methoxy-2-methyl isoflavone | Swisstarget prediction |
| CHRNA5 | gancao | 7-Methoxy-2-methyl isoflavone | Swisstarget prediction |
| SNCA | gancao | 7-Methoxy-2-methyl isoflavone | Swisstarget prediction |
| CPT1A | gancao | 7-Methoxy-2-methyl isoflavone | Swisstarget prediction |
| CCND1 | gancao | 7-Methoxy-2-methyl isoflavone | Swisstarget prediction |
| CCNE2 | gancao | 7-Methoxy-2-methyl isoflavone | Swisstarget prediction |
| PARP10 | gancao | 7-Methoxy-2-methyl isoflavone | Swisstarget prediction |
| BDKRB2 | gancao | 7-Methoxy-2-methyl isoflavone | Swisstarget prediction |
| PTGDR2 | gancao | 7-Methoxy-2-methyl isoflavone | Swisstarget prediction |
| SLC27A4 | gancao | 7-Methoxy-2-methyl isoflavone | Swisstarget prediction |
| NR1D1 | gancao | 7-Methoxy-2-methyl isoflavone | Swisstarget prediction |
| PPARA | gancao | formononetin | Swisstarget prediction |
| TLR9 | gancao | formononetin | Swisstarget prediction |
| PON1 | gancao | formononetin | Swisstarget prediction |
| DHODH | gancao | formononetin | Swisstarget prediction |
| STS | gancao | formononetin | Swisstarget prediction |
| ERCC5 | gancao | formononetin | Swisstarget prediction |
| FEN1 | gancao | formononetin | Swisstarget prediction |
| CDC7 | gancao | Calycosin | Swisstarget prediction |
| PLAT | gancao | Calycosin | Swisstarget prediction |
| SIRT2 | gancao | naringenin | Swisstarget prediction |
| CTSL | gancao | naringenin | Swisstarget prediction |
| CTSK | gancao | (2S)-2-[4-hydroxy-3-(3-methylbut-2-enyl)phenyl]-8,8-dimethyl-2,3-dihydropyrano[2,3-f]chromen-4-one | Swisstarget prediction |
| PDE7A | gancao | euchrenone | Swisstarget prediction |
| RPS6KB1 | gancao | euchrenone | Swisstarget prediction |
| MAPK8 | gancao | euchrenone | Swisstarget prediction |
| SGK1 | gancao | euchrenone | Swisstarget prediction |
| ADRA2A | gancao | euchrenone | Swisstarget prediction |
| ADRA2C | gancao | euchrenone | Swisstarget prediction |
| ADRA2B | gancao | euchrenone | Swisstarget prediction |
| KDM1A | gancao | euchrenone | Swisstarget prediction |
| NAAA | gancao | euchrenone | Swisstarget prediction |
| BMP1 | gancao | euchrenone | Swisstarget prediction |
| PITRM1 | gancao | euchrenone | Swisstarget prediction |
| EPHA5 | gancao | euchrenone | Swisstarget prediction |
| EPHA4 | gancao | euchrenone | Swisstarget prediction |
| EPHA8 | gancao | euchrenone | Swisstarget prediction |
| EPHA7 | gancao | euchrenone | Swisstarget prediction |
| EPHB3 | gancao | euchrenone | Swisstarget prediction |
| EPHB1 | gancao | euchrenone | Swisstarget prediction |
| EPHA1 | gancao | euchrenone | Swisstarget prediction |
| DRD3 | gancao | euchrenone | Swisstarget prediction |
| TNF | gancao | euchrenone | Swisstarget prediction |
| CAMK2D | gancao | euchrenone | Swisstarget prediction |
| PDGFRB | gancao | glyasperin B | Swisstarget prediction |
| GSK3A | gancao | glyasperin B | Swisstarget prediction |
| CASP6 | gancao | glyasperin B | Swisstarget prediction |
| TGFBR1 | gancao | glyasperin B | Swisstarget prediction |
| ACVR1 | gancao | glyasperin B | Swisstarget prediction |
| ADAMTS5 | gancao | glyasperin B | Swisstarget prediction |
| CDK1 | gancao | glyasperin B | Swisstarget prediction |
| ADAMTS4 | gancao | glyasperin B | Swisstarget prediction |
| PDK1 | gancao | glyasperin B | Swisstarget prediction |
| HDAC8 | gancao | glyasperin B | Swisstarget prediction |
| HDAC1 | gancao | glyasperin B | Swisstarget prediction |
| HDAC3 | gancao | glyasperin B | Swisstarget prediction |
| MARK1 | gancao | glyasperin B | Swisstarget prediction |
| PDGFRA | gancao | glyasperin B | Swisstarget prediction |
| NPY5R | gancao | glyasperin B | Swisstarget prediction |
| ENPP1 | gancao | glyasperin B | Swisstarget prediction |
| STAT6 | gancao | glyasperin B | Swisstarget prediction |
| FYN | gancao | glyasperin B | Swisstarget prediction |
| YES1 | gancao | glyasperin B | Swisstarget prediction |
| BLK | gancao | glyasperin B | Swisstarget prediction |
| CSK | gancao | glyasperin B | Swisstarget prediction |
| ROCK1 | gancao | glyasperin B | Swisstarget prediction |
| BMX | gancao | glyasperin B | Swisstarget prediction |
| SLC5A1 | gancao | glyasperin F | Swisstarget prediction |
| SPHK2 | gancao | glyasperin F | Swisstarget prediction |
| SPHK1 | gancao | glyasperin F | Swisstarget prediction |
| MAPK14 | gancao | glyasperin F | Swisstarget prediction |
| DPP4 | gancao | glyasperin F | Swisstarget prediction |
| HTR7 | gancao | glyasperin F | Swisstarget prediction |
| DPP7 | gancao | glyasperin F | Swisstarget prediction |
| DPP8 | gancao | glyasperin F | Swisstarget prediction |
| CSNK1G1 | gancao | glyasperin F | Swisstarget prediction |
| MAPK1 | gancao | glyasperin F | Swisstarget prediction |
| DUT | gancao | glyasperin F | Swisstarget prediction |
| EIF2AK2 | gancao | glyasperin F | Swisstarget prediction |
| MMP16 | gancao | glyasperin F | Swisstarget prediction |
| MMP15 | gancao | glyasperin F | Swisstarget prediction |
| MMP14 | gancao | glyasperin F | Swisstarget prediction |
| MMP26 | gancao | glyasperin F | Swisstarget prediction |
| GUSB | gancao | glyasperin F | Swisstarget prediction |
| TGM2 | gancao | glyasperin F | Swisstarget prediction |
| PARP2 | gancao | glyasperin F | Swisstarget prediction |
| NTRK1 | gancao | glyasperin F | Swisstarget prediction |
| ALOX15B | gancao | Glyasperin C | Swisstarget prediction |
| CTSD | gancao | Glyasperin C | Swisstarget prediction |
| ME1 | gancao | Glyasperin C | Swisstarget prediction |
| CMA1 | gancao | Glyasperin C | Swisstarget prediction |
| CCKBR | gancao | Glyasperin C | Swisstarget prediction |
| KIF11 | gancao | Glyasperin C | Swisstarget prediction |
| EPHX2 | gancao | Glyasperin C | Swisstarget prediction |
| GNRHR | gancao | Glyasperin C | Swisstarget prediction |
| ITK | gancao | Glyasperin C | Swisstarget prediction |
| PTGIR | gancao | Glyasperin C | Swisstarget prediction |
| PDGFRA | gancao | Glyasperin C | Swisstarget prediction |
| ADRA1A | gancao | Glyasperin C | Swisstarget prediction |
| ADRA1B | gancao | Glyasperin C | Swisstarget prediction |
| TRPV1 | gancao | Glyasperin C | Swisstarget prediction |
| NOX1 | gancao | Glyasperin C | Swisstarget prediction |
| SCARB1 | gancao | Glyasperin C | Swisstarget prediction |
| CHRM3 | gancao | Glyasperin C | Swisstarget prediction |
| TACR3 | gancao | Glyasperin C | Swisstarget prediction |
| TBXA2R | gancao | Glyasperin C | Swisstarget prediction |
| S1PR3 | gancao | Glyasperin C | Swisstarget prediction |
| S1PR1 | gancao | Glyasperin C | Swisstarget prediction |
| MELK | gancao | Glyasperin C | Swisstarget prediction |
| MDM4 | gancao | Glyasperin C | Swisstarget prediction |
| MDM2 | gancao | Glyasperin C | Swisstarget prediction |
| ICAM1 | gancao | Glyasperin C | Swisstarget prediction |
| ITGB2 | gancao | Glyasperin C | Swisstarget prediction |
| ITGAL | gancao | Glyasperin C | Swisstarget prediction |
| CHRM2 | gancao | Glyasperin C | Swisstarget prediction |
| P2RX3 | gancao | Glyasperin C | Swisstarget prediction |
| PTPN6 | gancao | Glyasperin C | Swisstarget prediction |
| CRHR1 | gancao | Isotrifoliol | Swisstarget prediction |
| IGFBP6 | gancao | Isotrifoliol | Swisstarget prediction |
| IGFBP4 | gancao | Isotrifoliol | Swisstarget prediction |
| IGFBP5 | gancao | Isotrifoliol | Swisstarget prediction |
| IGFBP2 | gancao | Isotrifoliol | Swisstarget prediction |
| IGFBP1 | gancao | Isotrifoliol | Swisstarget prediction |
| KCNA5 | gancao | Isotrifoliol | Swisstarget prediction |
| KCNA3 | gancao | Isotrifoliol | Swisstarget prediction |
| PTP4A3 | gancao | Isotrifoliol | Swisstarget prediction |
| GSR | gancao | Isotrifoliol | Swisstarget prediction |
| CYP1A2 | gancao | Isotrifoliol | Swisstarget prediction |
| MAPKAPK5 | gancao | (E)-1-(2,4-dihydroxyphenyl)-3-(2,2-dimethylchromen-6-yl)prop-2-en-1-one | Swisstarget prediction |
| NCOR2 | gancao | (E)-1-(2,4-dihydroxyphenyl)-3-(2,2-dimethylchromen-6-yl)prop-2-en-1-one | Swisstarget prediction |
| HDAC11 | gancao | (E)-1-(2,4-dihydroxyphenyl)-3-(2,2-dimethylchromen-6-yl)prop-2-en-1-one | Swisstarget prediction |
| HDAC10 | gancao | (E)-1-(2,4-dihydroxyphenyl)-3-(2,2-dimethylchromen-6-yl)prop-2-en-1-one | Swisstarget prediction |
| PRKCD | gancao | (E)-1-(2,4-dihydroxyphenyl)-3-(2,2-dimethylchromen-6-yl)prop-2-en-1-one | Swisstarget prediction |
| HPGDS | gancao | (E)-1-(2,4-dihydroxyphenyl)-3-(2,2-dimethylchromen-6-yl)prop-2-en-1-one | Swisstarget prediction |
| IKBKE | gancao | (E)-1-(2,4-dihydroxyphenyl)-3-(2,2-dimethylchromen-6-yl)prop-2-en-1-one | Swisstarget prediction |
| BACE2 | gancao | (E)-1-(2,4-dihydroxyphenyl)-3-(2,2-dimethylchromen-6-yl)prop-2-en-1-one | Swisstarget prediction |
| SF3B3 | gancao | (E)-1-(2,4-dihydroxyphenyl)-3-(2,2-dimethylchromen-6-yl)prop-2-en-1-one | Swisstarget prediction |
| NCOR1 | gancao | (E)-1-(2,4-dihydroxyphenyl)-3-(2,2-dimethylchromen-6-yl)prop-2-en-1-one | Swisstarget prediction |
| SLC9A1 | gancao | (E)-1-(2,4-dihydroxyphenyl)-3-(2,2-dimethylchromen-6-yl)prop-2-en-1-one | Swisstarget prediction |
| HIF1A | gancao | (E)-1-(2,4-dihydroxyphenyl)-3-(2,2-dimethylchromen-6-yl)prop-2-en-1-one | Swisstarget prediction |
| TAOK1 | gancao | (E)-1-(2,4-dihydroxyphenyl)-3-(2,2-dimethylchromen-6-yl)prop-2-en-1-one | Swisstarget prediction |
| TAOK3 | gancao | (E)-1-(2,4-dihydroxyphenyl)-3-(2,2-dimethylchromen-6-yl)prop-2-en-1-one | Swisstarget prediction |
| SREBF2 | gancao | (E)-1-(2,4-dihydroxyphenyl)-3-(2,2-dimethylchromen-6-yl)prop-2-en-1-one | Swisstarget prediction |
| CSF1R | gancao | (2S)-6-(2,4-dihydroxyphenyl)-2-(2-hydroxypropan-2-yl)-4-methoxy-2,3-dihydrofuro[3,2-g]chromen-7-one | Swisstarget prediction |
| SOAT1 | gancao | (2S)-6-(2,4-dihydroxyphenyl)-2-(2-hydroxypropan-2-yl)-4-methoxy-2,3-dihydrofuro[3,2-g]chromen-7-one | Swisstarget prediction |
| SOAT2 | gancao | (2S)-6-(2,4-dihydroxyphenyl)-2-(2-hydroxypropan-2-yl)-4-methoxy-2,3-dihydrofuro[3,2-g]chromen-7-one | Swisstarget prediction |
| KCNH2 | gancao | (2S)-6-(2,4-dihydroxyphenyl)-2-(2-hydroxypropan-2-yl)-4-methoxy-2,3-dihydrofuro[3,2-g]chromen-7-one | Swisstarget prediction |
| MGLL | gancao | (2S)-6-(2,4-dihydroxyphenyl)-2-(2-hydroxypropan-2-yl)-4-methoxy-2,3-dihydrofuro[3,2-g]chromen-7-one | Swisstarget prediction |
| ABHD6 | gancao | (2S)-6-(2,4-dihydroxyphenyl)-2-(2-hydroxypropan-2-yl)-4-methoxy-2,3-dihydrofuro[3,2-g]chromen-7-one | Swisstarget prediction |
| RARA | gancao | Semilicoisoflavone B | Swisstarget prediction |
| ADRB2 | gancao | Semilicoisoflavone B | Swisstarget prediction |
| ADRB1 | gancao | Semilicoisoflavone B | Swisstarget prediction |
| ERBB2 | gancao | Glepidotin A | Swisstarget prediction |
| GABRA3 | gancao | Glepidotin A | Swisstarget prediction |
| GABRA1 | gancao | Glepidotin A | Swisstarget prediction |
| CNR2 | gancao | Glepidotin A | Swisstarget prediction |
| DHFR | gancao | Glepidotin A | Swisstarget prediction |
| GRM4 | gancao | Phaseolinisoflavan | Swisstarget prediction |
| GABRB3 | gancao | Phaseolinisoflavan | Swisstarget prediction |
| GABRA6 | gancao | Phaseolinisoflavan | Swisstarget prediction |
| GABRG2 | gancao | Phaseolinisoflavan | Swisstarget prediction |
| LTA4H | gancao | Phaseolinisoflavan | Swisstarget prediction |
| ELANE | gancao | Phaseolinisoflavan | Swisstarget prediction |
| LRRK2 | gancao | Phaseolinisoflavan | Swisstarget prediction |
| GRK7 | gancao | Phaseolinisoflavan | Swisstarget prediction |
| HIPK4 | gancao | Phaseolinisoflavan | Swisstarget prediction |
| TAOK2 | gancao | Phaseolinisoflavan | Swisstarget prediction |
| PIK3C2G | gancao | Phaseolinisoflavan | Swisstarget prediction |
| PIP4K2C | gancao | Phaseolinisoflavan | Swisstarget prediction |
| FLT1 | gancao | Phaseolinisoflavan | Swisstarget prediction |
| CASK | gancao | Phaseolinisoflavan | Swisstarget prediction |
| PIP5K1C | gancao | Phaseolinisoflavan | Swisstarget prediction |
| DSTYK | gancao | Phaseolinisoflavan | Swisstarget prediction |
| FLT4 | gancao | Phaseolinisoflavan | Swisstarget prediction |
| MAP2K3 | gancao | Phaseolinisoflavan | Swisstarget prediction |
| PHKG2 | gancao | Phaseolinisoflavan | Swisstarget prediction |
| DAPK3 | gancao | Phaseolinisoflavan | Swisstarget prediction |
| CSNK1G2 | gancao | Phaseolinisoflavan | Swisstarget prediction |
| FGFR3 | gancao | Phaseolinisoflavan | Swisstarget prediction |
| JAK1 | gancao | Phaseolinisoflavan | Swisstarget prediction |
| PRKG2 | gancao | Phaseolinisoflavan | Swisstarget prediction |
| MAP2K4 | gancao | Phaseolinisoflavan | Swisstarget prediction |
| MAP2K2 | gancao | Phaseolinisoflavan | Swisstarget prediction |
| JAK2 | gancao | Phaseolinisoflavan | Swisstarget prediction |
| CDK7 | gancao | Phaseolinisoflavan | Swisstarget prediction |
| DAPK2 | gancao | Phaseolinisoflavan | Swisstarget prediction |
| RPS6KA4 | gancao | Phaseolinisoflavan | Swisstarget prediction |
| HTR1A | gancao | Glypallichalcone | Swisstarget prediction |
| MMP25 | gancao | Glypallichalcone | Swisstarget prediction |
| ADAM10 | gancao | Glypallichalcone | Swisstarget prediction |
| CDK8 | gancao | Glypallichalcone | Swisstarget prediction |
| CCNC | gancao | Glypallichalcone | Swisstarget prediction |
| FCER2 | gancao | Glypallichalcone | Swisstarget prediction |
| ADAM9 | gancao | Glypallichalcone | Swisstarget prediction |
| CXCR4 | gancao | Glypallichalcone | Swisstarget prediction |
| GLI2 | gancao | Licochalcone B | Swisstarget prediction |
| GLI1 | gancao | Licochalcone B | Swisstarget prediction |
| THRA | gancao | Licochalcone B | Swisstarget prediction |
| THRB | gancao | Licochalcone B | Swisstarget prediction |
| MAP4K4 | gancao | Licochalcone B | Swisstarget prediction |
| PRKCB | gancao | licochalcone G | Swisstarget prediction |
| TKT | gancao | licochalcone G | Swisstarget prediction |
| CNOT7 | gancao | Licoricone | Swisstarget prediction |
| OPRK1 | gancao | Licoricone | Swisstarget prediction |
| FBP1 | gancao | Gancaonin A | Swisstarget prediction |
| CALCA | gancao | Gancaonin A | Swisstarget prediction |
| KISS1R | gancao | Gancaonin A | Swisstarget prediction |
| PRKCE | gancao | Gancaonin B | Swisstarget prediction |
| HTR3A | gancao | Glycyrin | Swisstarget prediction |
| MERTK | gancao | Glycyrin | Swisstarget prediction |
| CTSB | gancao | Glycyrin | Swisstarget prediction |
| LIMK1 | gancao | Glycyrin | Swisstarget prediction |
| LIMK2 | gancao | Glycyrin | Swisstarget prediction |
| FKBP1A | gancao | Glycyrin | Swisstarget prediction |
| STK17B | gancao | Glycyrin | Swisstarget prediction |
| PRF1 | gancao | Glycyrin | Swisstarget prediction |
| RASGRP3 | gancao | Licocoumarone | Swisstarget prediction |
| HTR6 | gancao | Licocoumarone | Swisstarget prediction |
| ALOX5AP | gancao | Licocoumarone | Swisstarget prediction |
| NR3C2 | gancao | Licocoumarone | Swisstarget prediction |
| PSENEN | gancao | Licocoumarone | Swisstarget prediction |
| NCSTN | gancao | Licocoumarone | Swisstarget prediction |
| APH1A | gancao | Licocoumarone | Swisstarget prediction |
| PSEN1 | gancao | Licocoumarone | Swisstarget prediction |
| APH1B | gancao | Licocoumarone | Swisstarget prediction |
| PSEN2 | gancao | Licocoumarone | Swisstarget prediction |
| EDNRB | gancao | Licocoumarone | Swisstarget prediction |
| PI4KB | gancao | licoisoflavanone | Swisstarget prediction |
| PTK6 | gancao | licoisoflavanone | Swisstarget prediction |
| FGFR2 | gancao | licoisoflavanone | Swisstarget prediction |
| DBF4 | gancao | licoisoflavanone | Swisstarget prediction |
| ABAT | gancao | licoisoflavanone | Swisstarget prediction |
| BAD | gancao | shinpterocarpin | Swisstarget prediction |
| CLK3 | gancao | shinpterocarpin | Swisstarget prediction |
| NR4A1 | gancao | shinpterocarpin | Swisstarget prediction |
| PDE3A | gancao | shinpterocarpin | Swisstarget prediction |
| PRKCZ | gancao | (E)-3-[3,4-dihydroxy-5-(3-methylbut-2-enyl)phenyl]-1-(2,4-dihydroxyphenyl)prop-2-en-1-one | Swisstarget prediction |
| ILK | gancao | (E)-3-[3,4-dihydroxy-5-(3-methylbut-2-enyl)phenyl]-1-(2,4-dihydroxyphenyl)prop-2-en-1-one | Swisstarget prediction |
| QPCT | gancao | (E)-3-[3,4-dihydroxy-5-(3-methylbut-2-enyl)phenyl]-1-(2,4-dihydroxyphenyl)prop-2-en-1-one | Swisstarget prediction |
| AKT2 | gancao | (E)-3-[3,4-dihydroxy-5-(3-methylbut-2-enyl)phenyl]-1-(2,4-dihydroxyphenyl)prop-2-en-1-one | Swisstarget prediction |
| SLC5A4 | gancao | liquiritin | Swisstarget prediction |
| CDC42BPA | gancao | licopyranocoumarin | Swisstarget prediction |
| FKBP1A | gancao | licopyranocoumarin | Swisstarget prediction |
| WDR5 | gancao | licopyranocoumarin | Swisstarget prediction |
| WNT3A | gancao | licopyranocoumarin | Swisstarget prediction |
| ACVRL1 | gancao | licopyranocoumarin | Swisstarget prediction |
| PRSS1 | gancao | 3,22-Dihydroxy-11-oxo-delta(12)-oleanene-27-alpha-methoxycarbonyl-29-oic acid | Swisstarget prediction |
| CTRC | gancao | 3,22-Dihydroxy-11-oxo-delta(12)-oleanene-27-alpha-methoxycarbonyl-29-oic acid | Swisstarget prediction |
| EPAS1 | gancao | 3,22-Dihydroxy-11-oxo-delta(12)-oleanene-27-alpha-methoxycarbonyl-29-oic acid | Swisstarget prediction |
| HSD11B2 | gancao | 3,22-Dihydroxy-11-oxo-delta(12)-oleanene-27-alpha-methoxycarbonyl-29-oic acid | Swisstarget prediction |
| DNTT | gancao | 3,22-Dihydroxy-11-oxo-delta(12)-oleanene-27-alpha-methoxycarbonyl-29-oic acid | Swisstarget prediction |
| AVPR1A | gancao | 3,22-Dihydroxy-11-oxo-delta(12)-oleanene-27-alpha-methoxycarbonyl-29-oic acid | Swisstarget prediction |
| HCRTR2 | gancao | 3,22-Dihydroxy-11-oxo-delta(12)-oleanene-27-alpha-methoxycarbonyl-29-oic acid | Swisstarget prediction |
| HCRTR1 | gancao | 3,22-Dihydroxy-11-oxo-delta(12)-oleanene-27-alpha-methoxycarbonyl-29-oic acid | Swisstarget prediction |
| TACR2 | gancao | 3,22-Dihydroxy-11-oxo-delta(12)-oleanene-27-alpha-methoxycarbonyl-29-oic acid | Swisstarget prediction |
| PRKD1 | gancao | 3,22-Dihydroxy-11-oxo-delta(12)-oleanene-27-alpha-methoxycarbonyl-29-oic acid | Swisstarget prediction |
| PIK3C3 | gancao | 3,22-Dihydroxy-11-oxo-delta(12)-oleanene-27-alpha-methoxycarbonyl-29-oic acid | Swisstarget prediction |
| C5AR1 | gancao | 3,22-Dihydroxy-11-oxo-delta(12)-oleanene-27-alpha-methoxycarbonyl-29-oic acid | Swisstarget prediction |
| CCNT1 | gancao | 3,22-Dihydroxy-11-oxo-delta(12)-oleanene-27-alpha-methoxycarbonyl-29-oic acid | Swisstarget prediction |
| ACKR3 | gancao | 3,22-Dihydroxy-11-oxo-delta(12)-oleanene-27-alpha-methoxycarbonyl-29-oic acid | Swisstarget prediction |
| CXCR3 | gancao | 3,22-Dihydroxy-11-oxo-delta(12)-oleanene-27-alpha-methoxycarbonyl-29-oic acid | Swisstarget prediction |
| MAST3 | gancao | 3,22-Dihydroxy-11-oxo-delta(12)-oleanene-27-alpha-methoxycarbonyl-29-oic acid | Swisstarget prediction |
| VHL | gancao | 3,22-Dihydroxy-11-oxo-delta(12)-oleanene-27-alpha-methoxycarbonyl-29-oic acid | Swisstarget prediction |
| PLA2G7 | gancao | 3,22-Dihydroxy-11-oxo-delta(12)-oleanene-27-alpha-methoxycarbonyl-29-oic acid | Swisstarget prediction |
| PGGT1B | gancao | 3,22-Dihydroxy-11-oxo-delta(12)-oleanene-27-alpha-methoxycarbonyl-29-oic acid | Swisstarget prediction |
| EZH2 | gancao | 3,22-Dihydroxy-11-oxo-delta(12)-oleanene-27-alpha-methoxycarbonyl-29-oic acid | Swisstarget prediction |
| P2RY1 | gancao | 3,22-Dihydroxy-11-oxo-delta(12)-oleanene-27-alpha-methoxycarbonyl-29-oic acid | Swisstarget prediction |
| CASR | gancao | 3,22-Dihydroxy-11-oxo-delta(12)-oleanene-27-alpha-methoxycarbonyl-29-oic acid | Swisstarget prediction |
| REN | gancao | 3,22-Dihydroxy-11-oxo-delta(12)-oleanene-27-alpha-methoxycarbonyl-29-oic acid | Swisstarget prediction |
| CCR1 | gancao | 3,22-Dihydroxy-11-oxo-delta(12)-oleanene-27-alpha-methoxycarbonyl-29-oic acid | Swisstarget prediction |
| BMP4 | gancao | Glabridin | Swisstarget prediction |
| TNNT2 | gancao | Glabridin | Swisstarget prediction |
| TNNI3 | gancao | Glabridin | Swisstarget prediction |
| GRIN2B | gancao | Glabridin | Swisstarget prediction |
| TNNC1 | gancao | Glabridin | Swisstarget prediction |
| DRD2 | gancao | Glabridin | Swisstarget prediction |
| GRIN1 | gancao | Glabridin | Swisstarget prediction |
| MAP3K5 | gancao | Glabranin | Swisstarget prediction |
| GSTA1 | gancao | Glabranin | Swisstarget prediction |
| PTK2B | gancao | Glabranin | Swisstarget prediction |
| BCL2A1 | gancao | Glabranin | Swisstarget prediction |
| LYN | gancao | Glabranin | Swisstarget prediction |
| TXK | gancao | Glabranin | Swisstarget prediction |
| FGR | gancao | Glabranin | Swisstarget prediction |
| EPHA6 | gancao | Glabranin | Swisstarget prediction |
| TYRO3 | gancao | Glabranin | Swisstarget prediction |
| COQ8B | gancao | Glabranin | Swisstarget prediction |
| FAP | gancao | Glabranin | Swisstarget prediction |
| FADS1 | gancao | Glabranin | Swisstarget prediction |
| FPR1 | gancao | Glabranin | Swisstarget prediction |
| ANPEP | gancao | Glabranin | Swisstarget prediction |
| ASAH1 | gancao | Glabrene | Swisstarget prediction |
| DRD5 | gancao | Glabrene | Swisstarget prediction |
| RARG | gancao | Glabrene | Swisstarget prediction |
| HTR1B | gancao | Glabrene | Swisstarget prediction |
| TSPO | gancao | Glabrene | Swisstarget prediction |
| CYP2C19 | gancao | Glabrene | Swisstarget prediction |
| PHLPP2 | gancao | Glabrene | Swisstarget prediction |
| SLC2A1 | gancao | (-)-Medicocarpin | Swisstarget prediction |
| PRKCQ | gancao | HMO | Swisstarget prediction |
| CNR1 | gancao | HMO | Swisstarget prediction |
| LPAR6 | gancao | HMO | Swisstarget prediction |
| LPAR5 | gancao | HMO | Swisstarget prediction |
| ENPP2 | gancao | HMO | Swisstarget prediction |
| PREP | gancao | HMO | Swisstarget prediction |
| GPR119 | gancao | HMO | Swisstarget prediction |
| LPAR3 | gancao | HMO | Swisstarget prediction |
| LPAR2 | gancao | HMO | Swisstarget prediction |
| LPAR1 | gancao | HMO | Swisstarget prediction |
| LPAR4 | gancao | HMO | Swisstarget prediction |
| ESRRG | gancao | 3'-Hydroxy-4'-O-Methylglabridin | Swisstarget prediction |
| GRM1 | gancao | 3'-Hydroxy-4'-O-Methylglabridin | Swisstarget prediction |
| AHCY | gancao | 3'-Hydroxy-4'-O-Methylglabridin | Swisstarget prediction |
| ATM | gancao | 3'-Hydroxy-4'-O-Methylglabridin | Swisstarget prediction |
| ATR | gancao | 3'-Hydroxy-4'-O-Methylglabridin | Swisstarget prediction |
| ROCK2 | gancao | licochalcone a | Swisstarget prediction |
| MAPK10 | gancao | licochalcone a | Swisstarget prediction |
| CHRNA4 | gancao | licochalcone a | Swisstarget prediction |
| PRKACA | gancao | 3'-Methoxyglabridin | Swisstarget prediction |
| CBFB | gancao | 3'-Methoxyglabridin | Swisstarget prediction |
| NR1H3 | gancao | icos-5-enoic acid | Swisstarget prediction |
| G6PD | gancao | icos-5-enoic acid | Swisstarget prediction |
| CD81 | gancao | icos-5-enoic acid | Swisstarget prediction |
| MAPK3 | gancao | icos-5-enoic acid | Swisstarget prediction |
| RORA | gancao | icos-5-enoic acid | Swisstarget prediction |
| PTGDR | gancao | icos-5-enoic acid | Swisstarget prediction |
| CPT1B | gancao | icos-5-enoic acid | Swisstarget prediction |
| GIPR | gancao | icos-5-enoic acid | Swisstarget prediction |
| SLC22A6 | gancao | icos-5-enoic acid | Swisstarget prediction |
| SLC6A4 | gancao | icos-5-enoic acid | Swisstarget prediction |
| LDLR | gancao | Kanzonol F | Swisstarget prediction |
| DDX3X | gancao | Kanzonol F | Swisstarget prediction |
| CCNA2 | gancao | Kanzonol F | Swisstarget prediction |
| SLC2A3 | gancao | Kanzonol F | Swisstarget prediction |
| SLC2A2 | gancao | Kanzonol F | Swisstarget prediction |
| NOS1 | gancao | Kanzonol F | Swisstarget prediction |
| QPCTL | gancao | Kanzonol F | Swisstarget prediction |
| NOS3 | gancao | Kanzonol F | Swisstarget prediction |
| CCKAR | gancao | Kanzonol F | Swisstarget prediction |
| GPR55 | gancao | Kanzonol F | Swisstarget prediction |
| SLC8A1 | gancao | Kanzonol F | Swisstarget prediction |
| HTR2B | gancao | 7,2',4'-trihydroxy－5-methoxy-3－arylcoumarin | Swisstarget prediction |
| KDM5C | gancao | 7-Acetoxy-2-methylisoflavone | Swisstarget prediction |
| KDM4A | gancao | 7-Acetoxy-2-methylisoflavone | Swisstarget prediction |
| KDM4D | gancao | 7-Acetoxy-2-methylisoflavone | Swisstarget prediction |
| PIK3CD | gancao | 7-Acetoxy-2-methylisoflavone | Swisstarget prediction |
| PROKR1 | gancao | 7-Acetoxy-2-methylisoflavone | Swisstarget prediction |
| AGPAT2 | gancao | 7-Acetoxy-2-methylisoflavone | Swisstarget prediction |
| GRIN1 | gancao | 7-Acetoxy-2-methylisoflavone | Swisstarget prediction |
| GRIN2A | gancao | 7-Acetoxy-2-methylisoflavone | Swisstarget prediction |
| GRIA2 | gancao | 7-Acetoxy-2-methylisoflavone | Swisstarget prediction |
| CDK9 | gancao | 7-Acetoxy-2-methylisoflavone | Swisstarget prediction |
| SHH | gancao | 7-Acetoxy-2-methylisoflavone | Swisstarget prediction |
| KAT2B | gancao | 7-Acetoxy-2-methylisoflavone | Swisstarget prediction |
| KCNK3 | gancao | 7-Acetoxy-2-methylisoflavone | Swisstarget prediction |
| KCNK9 | gancao | 7-Acetoxy-2-methylisoflavone | Swisstarget prediction |
| TGFB1 | gancao | 7-Acetoxy-2-methylisoflavone | Swisstarget prediction |
| CDK9 | gancao | 7-Acetoxy-2-methylisoflavone | Swisstarget prediction |
| LTB4R | gancao | gadelaidic acid | Swisstarget prediction |
| TYMP | gancao | Vestitol | Swisstarget prediction |
| TAAR1 | gancao | Vestitol | Swisstarget prediction |
| FOLH1 | gancao | Gancaonin G | Swisstarget prediction |
| PAK4 | gancao | Gancaonin G | Swisstarget prediction |
| NAMPT | gancao | Gancaonin G | Swisstarget prediction |
| CFD | gancao | Licoagrocarpin | Swisstarget prediction |
| MARS | gancao | Licoagrocarpin | Swisstarget prediction |
| BRD4 | gancao | Licoagrocarpin | Swisstarget prediction |
| LDHA | gancao | Licoagrocarpin | Swisstarget prediction |
| PDE6C | gancao | Licoagrocarpin | Swisstarget prediction |
| ROS1 | gancao | Licoagrocarpin | Swisstarget prediction |
| ADAM33 | gancao | Licoagrocarpin | Swisstarget prediction |
| OXTR | gancao | Licoagrocarpin | Swisstarget prediction |
| PDF | gancao | Licoagrocarpin | Swisstarget prediction |
| SMARCA2 | gancao | Licoagrocarpin | Swisstarget prediction |
| STAT3 | gancao | Licoagrocarpin | Swisstarget prediction |
| PTGFR | gancao | 18α-hydroxyglycyrrhetic acid | Swisstarget prediction |
| ITGAL | gancao | 18α-hydroxyglycyrrhetic acid | Swisstarget prediction |
| NR1I2 | gancao | 18α-hydroxyglycyrrhetic acid | Swisstarget prediction |
| PDE2A | gancao | 18α-hydroxyglycyrrhetic acid | Swisstarget prediction |
| F11 | gancao | 18α-hydroxyglycyrrhetic acid | Swisstarget prediction |
| S1PR2 | gancao | 18α-hydroxyglycyrrhetic acid | Swisstarget prediction |
| OAT | gancao | 18α-hydroxyglycyrrhetic acid | Swisstarget prediction |
| SLC10A2 | gancao | 18α-hydroxyglycyrrhetic acid | Swisstarget prediction |
| ITGB3 | gancao | 18α-hydroxyglycyrrhetic acid | Swisstarget prediction |
| ITGA4 | gancao | 18α-hydroxyglycyrrhetic acid | Swisstarget prediction |
| ITGAV | gancao | 18α-hydroxyglycyrrhetic acid | Swisstarget prediction |
| ITGA2B | gancao | 18α-hydroxyglycyrrhetic acid | Swisstarget prediction |
| ITGB1 | gancao | 18α-hydroxyglycyrrhetic acid | Swisstarget prediction |
| CTSA | gancao | 18α-hydroxyglycyrrhetic acid | Swisstarget prediction |
| NR1H2 | shengjiang | poriferast-5-en-26beta-ol | Swisstarget prediction |
| DHCR7 | shengjiang | poriferast-5-en-29beta-ol | Swisstarget prediction |
| SLC6A11 | shengjiang | Dihydrocapsaicin | Swisstarget prediction |
| SLC6A13 | shengjiang | Dihydrocapsaicin | Swisstarget prediction |
| CHRM4 | shengjiang | Dihydrocapsaicin | Swisstarget prediction |
| HRH2 | shengjiang | Dihydrocapsaicin | Swisstarget prediction |
| HRH1 | shengjiang | Dihydrocapsaicin | Swisstarget prediction |
| NPY1R | shengjiang | Dihydrocapsaicin | Swisstarget prediction |
| CHRM5 | shengjiang | Dihydrocapsaicin | Swisstarget prediction |
| LIPG | shengjiang | Dihydrocapsaicin | Swisstarget prediction |
| CCR8 | shengjiang | Dihydrocapsaicin | Swisstarget prediction |
| TNK2 | shengjiang | Dihydrocapsaicin | Swisstarget prediction |
| DMPK | shengjiang | Dihydrocapsaicin | Swisstarget prediction |
| PKN2 | shengjiang | Dihydrocapsaicin | Swisstarget prediction |
| F9 | shengjiang | Dihydrocapsaicin | Swisstarget prediction |
| NFE2L2 | shengjiang | curcumin | Swisstarget prediction |
| CHUK | shengjiang | curcumin | Swisstarget prediction |
| IKBKG | shengjiang | curcumin | Swisstarget prediction |
| GRIK1 | shengjiang | curcumin | Swisstarget prediction |
| CELA1 | shengjiang | curcumin | Swisstarget prediction |
| LYPLA1 | shengjiang | gingerol | Swisstarget prediction |
| LYPLA2 | shengjiang | gingerol | Swisstarget prediction |
| POLA1 | shengjiang | gingerol | Swisstarget prediction |
| ST6GAL1 | shengjiang | gingerol | Swisstarget prediction |
| KCNJ1 | shengjiang | gingerol | Swisstarget prediction |
| ADA | shengjiang | gingerol | Swisstarget prediction |
| PER2 | shengjiang | gingerol | Swisstarget prediction |
| BRD2 | shengjiang | gingerol | Swisstarget prediction |
| BRD3 | shengjiang | gingerol | Swisstarget prediction |
| SORD | shengjiang | gingerol | Swisstarget prediction |
| TYK2 | shengjiang | gingerol | Swisstarget prediction |
| MKNK2 | shengjiang | gingerol | Swisstarget prediction |
| MAP3K7 | shengjiang | gingerol | Swisstarget prediction |
| TAB1 | shengjiang | gingerol | Swisstarget prediction |
| PIK3R1 | shengjiang | gingerol | Swisstarget prediction |
| PRKX | dazao | stepharine | Swisstarget prediction |
| HTR1F | dazao | stepharine | Swisstarget prediction |
| MC4R | dazao | stepharine | Swisstarget prediction |
| TTK | dazao | stepharine | Swisstarget prediction |
| PARP3 | dazao | stepharine | Swisstarget prediction |
| DPP9 | dazao | stepharine | Swisstarget prediction |
| CDC42 | dazao | stepharine | Swisstarget prediction |
| MALT1 | dazao | stepharine | Swisstarget prediction |
| PLK4 | dazao | coumestrol | Swisstarget prediction |
| TEK | dazao | coumestrol | Swisstarget prediction |
| PRCP | dazao | Mauritine D | Swisstarget prediction |
| NPY2R | dazao | Mauritine D | Swisstarget prediction |
| MC5R | dazao | Mauritine D | Swisstarget prediction |
| KCNJ3 | dazao | Mauritine D | Swisstarget prediction |
| KCNJ5 | dazao | Mauritine D | Swisstarget prediction |
| OPRL1 | dazao | Mauritine D | Swisstarget prediction |
| CCR3 | dazao | Mauritine D | Swisstarget prediction |
| HTR1D | dazao | Mauritine D | Swisstarget prediction |
| BIRC8 | dazao | Mauritine D | Swisstarget prediction |
| PDE11A | dazao | Mauritine D | Swisstarget prediction |
| HCN4 | dazao | Mauritine D | Swisstarget prediction |
| HCN1 | dazao | Mauritine D | Swisstarget prediction |
| ACACA | dazao | Mauritine D | Swisstarget prediction |
| OXSR1 | dazao | Mauritine D | Swisstarget prediction |
| STK39 | dazao | Mauritine D | Swisstarget prediction |
| MAP3K13 | dazao | Mauritine D | Swisstarget prediction |
| ICK | dazao | Mauritine D | Swisstarget prediction |
| MAP3K15 | dazao | Mauritine D | Swisstarget prediction |
| MAST1 | dazao | Mauritine D | Swisstarget prediction |
| SBK1 | dazao | Mauritine D | Swisstarget prediction |
| PRPF4B | dazao | Mauritine D | Swisstarget prediction |
| MAP3K12 | dazao | Mauritine D | Swisstarget prediction |
| PRKAA2 | dazao | Mauritine D | Swisstarget prediction |
| ADRA1D | dazao | Stepholidine | Swisstarget prediction |
| ADRB3 | dazao | Stepholidine | Swisstarget prediction |
| CHRNB4 | dazao | Stepholidine | Swisstarget prediction |
| CHRNA2 | dazao | Stepholidine | Swisstarget prediction |
| CHRNA3 | dazao | Stepholidine | Swisstarget prediction |
| CHRNB4 | dazao | Stepholidine | Swisstarget prediction |
| RBBP9 | dazao | Stepholidine | Swisstarget prediction |
| KCNN1 | dazao | Stepholidine | Swisstarget prediction |
| KCNN3 | dazao | Stepholidine | Swisstarget prediction |
| KCNN2 | dazao | Stepholidine | Swisstarget prediction |
| DYRK2 | dazao | Stepholidine | Swisstarget prediction |
| JUN | dazao | Stepholidine | Swisstarget prediction |
| PDE1A | dazao | Stepholidine | Swisstarget prediction |
| PTPRCAP | dazao | Stepholidine | Swisstarget prediction |
| HTR5A | dazao | Nuciferin | Swisstarget prediction |
| TH | dazao | Nuciferin | Swisstarget prediction |
| CHRNA6 | dazao | Nuciferin | Swisstarget prediction |
| CHRNA3 | dazao | Nuciferin | Swisstarget prediction |
| CHRNA1 | dazao | Nuciferin | Swisstarget prediction |
| CHRNB3 | dazao | Nuciferin | Swisstarget prediction |
| HRH4 | dazao | Nuciferin | Swisstarget prediction |
| HTR1E | dazao | Nuciferin | Swisstarget prediction |
| SCN4A | dazao | Nuciferin | Swisstarget prediction |
| BIRC2 | dazao | Nuciferin | Swisstarget prediction |
| CHRNG | dazao | Nuciferin | Swisstarget prediction |
| CHRND | dazao | Nuciferin | Swisstarget prediction |
| CHRNB1 | dazao | Nuciferin | Swisstarget prediction |
| HASPIN | dazao | Nuciferin | Swisstarget prediction |
| GRB2 | dazao | Protoporphyrin | Swisstarget prediction |
| PDE3B | dazao | Protoporphyrin | Swisstarget prediction |
| MLNR | dazao | Protoporphyrin | Swisstarget prediction |
| ITGAV | dazao | Protoporphyrin | Swisstarget prediction |
| ITGB6 | dazao | Protoporphyrin | Swisstarget prediction |
| ITGB5 | dazao | Protoporphyrin | Swisstarget prediction |
| PTPRC | dazao | Protoporphyrin | Swisstarget prediction |
| CASP8 | dazao | Protoporphyrin | Swisstarget prediction |
| CASP1 | dazao | Protoporphyrin | Swisstarget prediction |
| CSNK2A2 | dazao | Protoporphyrin | Swisstarget prediction |
| P2RY12 | dazao | Protoporphyrin | Swisstarget prediction |
| PGA5 | dazao | Protoporphyrin | Swisstarget prediction |
| ZAP70 | dazao | Protoporphyrin | Swisstarget prediction |
| KARS | dazao | Protoporphyrin | Swisstarget prediction |
| TRHR | dazao | Fumarine | Swisstarget prediction |
| SIRT3 | dazao | Fumarine | Swisstarget prediction |
| TRAP1 | dazao | Moupinamide | Swisstarget prediction |
| RBP4 | dazao | beta-carotene | Swisstarget prediction |
| GRIK2 | dazao | oleanolic acid | Swisstarget prediction |
| HSP90AA2P | guizhi | (-)-taxifolin | TCMSP |
| DPEP1 | guizhi | (+)-catechin | TCMSP |
| NCOA2 | guizhi | (+)-catechin | TCMSP |
| CALML3 | guizhi | (+)-catechin | TCMSP |
| CAT | guizhi | (+)-catechin | TCMSP |
| HAS2 | guizhi | (+)-catechin | TCMSP |
| DGAT2 | guizhi | taxifolin | TCMSP |
| MTTP | guizhi | taxifolin | TCMSP |
| APOB | guizhi | taxifolin | TCMSP |
| SCN5A | gancao | kanzonols W | TCMSP |
| NCOA1 | gancao | kanzonols W | TCMSP |
| F7 | gancao | Glepidotin B | TCMSP |
| IGHG1 | gancao | Glepidotin B | TCMSP |
| PKIA | gancao | (2R)-7-hydroxy-2-(4-hydroxyphenyl)chroman-4-one | TCMSP |
| RXRB | gancao | 2-[(3R)-8,8-dimethyl-3,4-dihydro-2H-pyrano[6,5-f]chromen-3-yl]-5-methoxyphenol | TCMSP |
| PTGS2 | shengjiang | alpha-Curcumene | TCMSP |
| COX2 | shengjiang | alpha-Curcumene | TCMSP |
| SLC6A2 | shengjiang | alpha-Curcumene | TCMSP |
| NET1 | shengjiang | alpha-Curcumene | TCMSP |
| SLC6A5 | shengjiang | alpha-Curcumene | TCMSP |
| ADRA1A | shengjiang | alpha-Curcumene | TCMSP |
| ADRA1C | shengjiang | alpha-Curcumene | TCMSP |
| SLC6A3 | shengjiang | alpha-Curcumene | TCMSP |
| DAT1 | shengjiang | alpha-Curcumene | TCMSP |
| HGF | shaoyao | benzoyl paeoniflorin | Genecard |
| BAX | shaoyao | benzoyl paeoniflorin | Genecard |
| RHOA | shaoyao | benzoyl paeoniflorin | Genecard |
| FASLG | shaoyao | benzoyl paeoniflorin | Genecard |
| FAS | shaoyao | benzoyl paeoniflorin | Genecard |
| CCN1 | shaoyao | benzoyl paeoniflorin | Genecard |
| GPD1 | dazao | zizyphus saponin 2 | Genecard |
