# Supplementary material for: To Predict Anti-Inflammatory and Immunomodulatory Targets of Guizhi Decoction in Treating Asthma Based on Network Pharmacology, Molecular Docking, and Experimental Validation
Source: Evid Based Complement Alternat Med. 2021 Dec 20;2021:9033842. doi: 10.1155/2021/9033842 (PMC8712140; doi:10.1155/2021/9033842)
Supplement: Supplementary Materials — Supplementary Material Table S1: 134 active compounds from TCMSP database and literature in Guizhi Decoction. Supplementary Material Table S2: drug targets information of different ingredients in Guizhi Decoction. Supplementary Material Table S3: target information at the intersection of drug targets and disease targets. Supplementary Material Table S4: core gene information filtered according to the “betweenness,” “closeness,” and “degree” values. Supplementary Material Table S5: details of the known ligand of the top targets. [file 9033842.f1.zip › 9033842.f1/Supplementary Material Table S3 (1).docx]

***Supplementary Material***

| **Table S3** Target information at the intersection of drug targets and disease targets | | |
| --- | --- | --- |
| **Gene** | **Herb** | **MolName** |
| CHUK | dazao | beta-sitosterol |
| SLC9A1 | dazao | beta-sitosterol |
| RORA | dazao | beta-sitosterol |
| GABBR1 | dazao | beta-sitosterol |
| HTR1A | dazao | beta-sitosterol |
| NR3C1 | dazao | coumestrol |
| PDE4B | dazao | coumestrol |
| NOS1 | dazao | coumestrol |
| GLI2 | dazao | coumestrol |
| SNCA | dazao | coumestrol |
| GRM1 | dazao | coumestrol |
| TGFB1 | dazao | Fumarine |
| MAP3K7 | dazao | Fumarine |
| TRPV1 | dazao | Mairin |
| PDE4A | dazao | Mauritine D |
| PARP1 | dazao | Mauritine D |
| GRK5 | dazao | Mauritine D |
| MAPK14 | dazao | Mauritine D |
| MB | dazao | Mauritine D |
| REN | dazao | Mauritine D |
| HMGCR | dazao | Mauritine D |
| ERCC5 | dazao | Mauritine D |
| MMP7 | dazao | Mauritine D |
| NOX1 | dazao | Mauritine D |
| G6PD | dazao | Moupinamide |
| SHH | dazao | Moupinamide |
| SRC | dazao | Moupinamide |
| IRAK4 | dazao | Moupinamide |
| PTGER3 | dazao | Moupinamide |
| JAK1 | dazao | Moupinamide |
| F3 | dazao | Nuciferin |
| PRKDC | dazao | Nuciferin |
| GCGR | dazao | Nuciferin |
| NR1H2 | dazao | Nuciferin |
| ESRRA | dazao | Nuciferin |
| MMP1 | dazao | oleanolic acid |
| FLT3 | dazao | oleanolic acid |
| OPRD1 | dazao | oleanolic acid |
| MMP14 | dazao | oleanolic acid |
| NFE2L2 | dazao | Protoporphyrin |
| STAT3 | dazao | Protoporphyrin |
| RARA | dazao | Protoporphyrin |
| TYK2 | dazao | Protoporphyrin |
| PDE3A | dazao | quercetin |
| PLAU | dazao | quercetin |
| KIT | dazao | quercetin |
| FLT1 | dazao | quercetin |
| DRD3 | dazao | quercetin |
| PRKCQ | dazao | quercetin |
| EDNRB | dazao | quercetin |
| PTGER2 | dazao | stepharine |
| HDAC7 | dazao | stepharine |
| ADRB3 | dazao | Stepholidine |
| PLA2G7 | dazao | Stepholidine |
| GAPDH | dazao | Stepholidine |
| RHOA | dazao | Stepholidine |
| MPO | dazao | Stigmasterol |
| ITK | dazao | Stigmasterol |
| C5AR1 | dazao | Stigmasterol |
| NAMPT | dazao | Stigmasterol |
| MAP3K9 | dazao | Stigmasterol |
| SERPINA6 | dazao | ursolic acid |
| SLC6A3 | dazao | ursolic acid |
| SLC6A2 | dazao | ursolic acid |
| TNF | dazao | ursolic acid |
| UGT2B7 | dazao | ursolic acid |
| PTGDR | dazao | ursolic acid |
| PTGS2 | dazao | ursolic acid |
| STAT6 | dazao | ursolic acid |
| TLR4 | dazao | ursolic acid |
| GSTP1 | dazao | ursolic acid |
| PTGDR2 | dazao | ursolic acid |
| VDR | dazao | ursolic acid |
| TLR9 | dazao | ursolic acid |
| MMP2 | dazao | ursolic acid |
| PLA2G2A | dazao | ursolic acid |
| PIK3CG | dazao | ursolic acid |
| ITGAL | dazao | ursolic acid |
| TACR2 | dazao | ursolic acid |
| PDGFRA | dazao | ursolic acid |
| VEGFA | dazao | ursolic acid |
| TGFBR1 | dazao | ursolic acid |
| SCN4A | dazao | ursolic acid |
| TXK | dazao | ursolic acid |
| PRKCZ | dazao | ursolic acid |
| LYN | dazao | ursolic acid |
| TERT | dazao | ursolic acid |
| TTR | dazao | ursolic acid |
| STS | dazao | ursolic acid |
| XPO1 | dazao | ursolic acid |
| PTPRC | dazao | ursolic acid |
| NTRK1 | dazao | ursolic acid |
| TRPA1 | dazao | ursolic acid |
| NRAS | dazao | ursolic acid |
| TOP2A | dazao | ursolic acid |
| SMARCA2 | dazao | ursolic acid |
| SLC6A13 | dazao | ursolic acid |
| TSPO | dazao | ursolic acid |
| PIK3CD | dazao | ursolic acid |
| TKT | dazao | ursolic acid |
| PLG | dazao | ursolic acid |
| TNNI3 | dazao | ursolic acid |
| PTK2 | dazao | ursolic acid |
| SCN5A | dazao | ursolic acid |
| TBK1 | dazao | ursolic acid |
| VCP | dazao | ursolic acid |
| SCN9A | dazao | ursolic acid |
| TUBB1 | dazao | ursolic acid |
| PTK6 | dazao | ursolic acid |
| PRSS1 | dazao | ursolic acid |
| XDH | dazao | ursolic acid |
| TRPM8 | dazao | ursolic acid |
| NCOA1 | dazao | ursolic acid |
| PRKCE | dazao | ursolic acid |
| TEK | dazao | ursolic acid |
| TNKS | dazao | ursolic acid |
| PTAFR | dazao | ursolic acid |
| ZAP70 | dazao | ursolic acid |
| TH | dazao | ursolic acid |
| TAAR1 | dazao | ursolic acid |
| RELA | dazao | ursolic acid |
| TYRO3 | dazao | ursolic acid |
| PLAT | dazao | ursolic acid |
| F10 | dazao | ursolic acid |
| XIAP | dazao | ursolic acid |
| PIM2 | dazao | ursolic acid |
| IGHG1 | dazao | ursolic acid |
| THRB | dazao | ursolic acid |
| SLC8A1 | dazao | ursolic acid |
| PLA2G1B | dazao | ursolic acid |
| TBXAS1 | dazao | ursolic acid |
| TACR3 | dazao | ursolic acid |
| PSENEN | dazao | ursolic acid |
| PSEN1 | dazao | ursolic acid |
| CXCR3 | gancao | (-)-Medicocarpin |
| BCL2 | gancao | (2S)-6-(2,4-dihydroxyphenyl)-2-(2-hydroxypropan-2-yl)-4-methoxy-2,3-dihydrofuro[3,2-g]chromen-7-one |
| ADORA3 | gancao | (E)-1-(2,4-dihydroxyphenyl)-3-(2,2-dimethylchromen-6-yl)prop-2-en-1-one |
| ABCG2 | gancao | (E)-1-(2,4-dihydroxyphenyl)-3-(2,2-dimethylchromen-6-yl)prop-2-en-1-one |
| CYP11B1 | gancao | (E)-1-(2,4-dihydroxyphenyl)-3-(2,2-dimethylchromen-6-yl)prop-2-en-1-one |
| ALOX15 | gancao | (E)-1-(2,4-dihydroxyphenyl)-3-(2,2-dimethylchromen-6-yl)prop-2-en-1-one |
| MAPK3 | gancao | (E)-1-(2,4-dihydroxyphenyl)-3-(2,2-dimethylchromen-6-yl)prop-2-en-1-one |
| PTPN22 | gancao | (E)-1-(2,4-dihydroxyphenyl)-3-(2,2-dimethylchromen-6-yl)prop-2-en-1-one |
| LGALS9 | gancao | (E)-1-(2,4-dihydroxyphenyl)-3-(2,2-dimethylchromen-6-yl)prop-2-en-1-one |
| LTA4H | gancao | (E)-3-[3,4-dihydroxy-5-(3-methylbut-2-enyl)phenyl]-1-(2,4-dihydroxyphenyl)prop-2-en-1-one |
| HCRTR1 | gancao | (E)-3-[3,4-dihydroxy-5-(3-methylbut-2-enyl)phenyl]-1-(2,4-dihydroxyphenyl)prop-2-en-1-one |
| SLC6A4 | gancao | 18α-hydroxyglycyrrhetic acid |
| HSPA5 | gancao | 18α-hydroxyglycyrrhetic acid |
| PTGES | gancao | 18α-hydroxyglycyrrhetic acid |
| LTB4R | gancao | 1-Methoxyphaseollidin |
| FEN1 | gancao | 1-Methoxyphaseollidin |
| CFD | gancao | 1-Methoxyphaseollidin |
| ANPEP | gancao | 1-Methoxyphaseollidin |
| CTSK | gancao | 1-Methoxyphaseollidin |
| APH1B | gancao | 1-Methoxyphaseollidin |
| SYK | gancao | 3,22-Dihydroxy-11-oxo-delta(12)-oleanene-27-alpha-methoxycarbonyl-29-oic acid |
| CBR1 | gancao | 3,22-Dihydroxy-11-oxo-delta(12)-oleanene-27-alpha-methoxycarbonyl-29-oic acid |
| ACKR3 | gancao | 3,22-Dihydroxy-11-oxo-delta(12)-oleanene-27-alpha-methoxycarbonyl-29-oic acid |
| HRH1 | gancao | 3'-Hydroxy-4'-O-Methylglabridin |
| GRIN2B | gancao | 3'-Hydroxy-4'-O-Methylglabridin |
| AKR1C4 | gancao | 3'-Hydroxy-4'-O-Methylglabridin |
| SHBG | gancao | 3'-Methoxyglabridin |
| EDNRA | gancao | 3'-Methoxyglabridin |
| PDGFRB | gancao | 7,2',4'-trihydroxy－5-methoxy-3－arylcoumarin |
| MDM2 | gancao | 7,2',4'-trihydroxy－5-methoxy-3－arylcoumarin |
| CMA1 | gancao | 7-Acetoxy-2-methylisoflavone |
| PTGER4 | gancao | 7-Acetoxy-2-methylisoflavone |
| SERPINE1 | gancao | 7-Acetoxy-2-methylisoflavone |
| EPHX1 | gancao | 7-Acetoxy-2-methylisoflavone |
| PDE10A | gancao | 7-Acetoxy-2-methylisoflavone |
| BDKRB2 | gancao | 7-Methoxy-2-methyl isoflavone |
| CYP2A6 | gancao | 7-Methoxy-2-methyl isoflavone |
| MAOB | gancao | DFV |
| FGF2 | gancao | DFV |
| CSK | gancao | DFV |
| BLK | gancao | DFV |
| CYP17A1 | gancao | euchrenone |
| FASLG | gancao | euchrenone |
| EP300 | gancao | euchrenone |
| HRH4 | gancao | euchrenone |
| KDM4C | gancao | euchrenone |
| DRD4 | gancao | euchrenone |
| CASR | gancao | euchrenone |
| GSTA1 | gancao | euchrenone |
| EPHX2 | gancao | euchrenone |
| HSD11B2 | gancao | formononetin |
| COMT | gancao | formononetin |
| ALOX15B | gancao | formononetin |
| JAK3 | gancao | formononetin |
| RORC | gancao | gadelaidic acid |
| PRKCA | gancao | gadelaidic acid |
| IGFBP1 | gancao | gadelaidic acid |
| GRK7 | gancao | Gancaonin A |
| FAS | gancao | Gancaonin B |
| MAPT | gancao | Gancaonin B |
| HTR2A | gancao | Gancaonin G |
| ENPP1 | gancao | Gancaonin G |
| BCL2A1 | gancao | Glabranin |
| ADRA2B | gancao | Glabrene |
| ADORA2B | gancao | Glabrene |
| SELP | gancao | Glabrene |
| MMP3 | gancao | Glabrene |
| HRAS | gancao | Glabrene |
| HSD11B1 | gancao | Glabridin |
| NR3C2 | gancao | Glabridin |
| LGALS3 | gancao | Glabridin |
| PTGER1 | gancao | Glabridin |
| ESRRB | gancao | Glabridin |
| MAPK1 | gancao | Glabrone |
| IGFBP3 | gancao | Glepidotin A |
| GBA | gancao | Glepidotin A |
| BAX | gancao | Glepidotin A |
| CCKBR | gancao | Glepidotin A |
| KCNMA1 | gancao | Glepidotin A |
| NFKB1 | gancao | glyasperin B |
| MTOR | gancao | glyasperin B |
| KDM1A | gancao | glyasperin B |
| PON1 | gancao | glyasperin B |
| HDAC9 | gancao | glyasperin B |
| ACVR1 | gancao | glyasperin B |
| IGFBP4 | gancao | glyasperin B |
| ADAMTS4 | gancao | glyasperin B |
| ADRA1A | gancao | Glyasperin C |
| FPR1 | gancao | Glyasperin C |
| EGFR | gancao | Glyasperin C |
| PTGFR | gancao | Glyasperin C |
| MGAM | gancao | Glyasperin C |
| PFKFB3 | gancao | glyasperin F |
| MC4R | gancao | glyasperin F |
| IDO1 | gancao | Glycyrin |
| IDH1 | gancao | Glycyrin |
| GABRA6 | gancao | Glycyrin |
| ECE1 | gancao | Glycyrin |
| FGR | gancao | Glycyrin |
| CHRM4 | gancao | Glycyrol |
| CFTR | gancao | Glycyrol |
| CCND1 | gancao | Glycyrol |
| GRK2 | gancao | Glycyrrhiza flavonol A |
| HDAC2 | gancao | Glypallichalcone |
| FGFR2 | gancao | Glypallichalcone |
| IKBKB | gancao | Glypallichalcone |
| ADAM9 | gancao | Glypallichalcone |
| CTSL | gancao | Glypallichalcone |
| CTSB | gancao | Glypallichalcone |
| CTSS | gancao | HMO |
| NOX4 | gancao | HMO |
| FYN | gancao | HMO |
| MMP8 | gancao | icos-5-enoic acid |
| ITGB3 | gancao | icos-5-enoic acid |
| ADRB2 | gancao | Inermine |
| ITGAV | gancao | Inermine |
| CD38 | gancao | Inermine |
| CHRNA7 | gancao | Inermine |
| IL2 | gancao | Isoglycyrol |
| PTGIR | gancao | Isoglycyrol |
| NOS2 | gancao | Isolicoflavonol |
| ARG1 | gancao | Isolicoflavonol |
| HRH2 | gancao | Isolicoflavonol |
| DPP4 | gancao | Isolicoflavonol |
| PDE3B | gancao | Isolicoflavonol |
| CCR1 | gancao | Isolicoflavonol |
| CA2 | gancao | Isolicoflavonol |
| PDPK1 | gancao | Isolicoflavonol |
| FADS1 | gancao | Isolicoflavonol |
| MMP9 | gancao | isorhamnetin |
| MMP12 | gancao | isorhamnetin |
| HDAC8 | gancao | isorhamnetin |
| CSF1R | gancao | isorhamnetin |
| CYP1A2 | gancao | Isotrifoliol |
| CYP2C9 | gancao | Isotrifoliol |
| PTGS1 | gancao | Isotrifoliol |
| OPRM1 | gancao | Isotrifoliol |
| F2 | gancao | Isotrifoliol |
| HDAC5 | gancao | Isotrifoliol |
| GUSB | gancao | Isotrifoliol |
| CHRM2 | gancao | Jaranol |
| CHRM5 | gancao | Jaranol |
| HTR3A | gancao | Jaranol |
| CDK2 | gancao | Jaranol |
| CASP3 | gancao | Jaranol |
| AHR | gancao | Jaranol |
| MAP4K4 | gancao | Jaranol |
| CDC25A | gancao | Jaranol |
| EZR | gancao | kaempferol |
| ERBB2 | gancao | kaempferol |
| BRAF | gancao | kaempferol |
| ALOX12 | gancao | kaempferol |
| GRK3 | gancao | Kanzonol F |
| ADAM33 | gancao | Licoagrocarpin |
| KDR | gancao | Licoagrocarpin |
| CCKAR | gancao | Licoagrocarpin |
| MAPK10 | gancao | Licoagrocarpin |
| MCL1 | gancao | Licoagrocarpin |
| MIF | gancao | Licoagroisoflavone |
| PRKAA2 | gancao | Licoagroisoflavone |
| PLA2G10 | gancao | Licoagroisoflavone |
| SGK1 | gancao | Licoagroisoflavone |
| ITGA4 | gancao | licochalcone a |
| MET | gancao | licochalcone a |
| ESRRG | gancao | licochalcone a |
| CYP2D6 | gancao | Licochalcone B |
| ITGB2 | gancao | Licochalcone B |
| PNMT | gancao | Licochalcone B |
| DHODH | gancao | Licochalcone B |
| CES2 | gancao | Licochalcone B |
| CYP2C19 | gancao | licochalcone G |
| PDE5A | gancao | licochalcone G |
| DRD2 | gancao | licochalcone G |
| HAO1 | gancao | licochalcone G |
| HSP90AB1 | gancao | licochalcone G |
| ALOX5AP | gancao | Licocoumarone |
| APH1A | gancao | Licocoumarone |
| MYLK | gancao | licoisoflavanone |
| HPGDS | gancao | licoisoflavanone |
| PGF | gancao | licoisoflavanone |
| ABAT | gancao | licoisoflavanone |
| HTR1B | gancao | licoisoflavanone |
| HSD3B1 | gancao | licoisoflavanone |
| NAT1 | gancao | licoisoflavanone |
| KAT2B | gancao | Licoisoflavone B |
| NR1I2 | gancao | licopyranocoumarin |
| HSPA8 | gancao | licopyranocoumarin |
| ACVRL1 | gancao | licopyranocoumarin |
| ELANE | gancao | Licoricone |
| HIF1A | gancao | Licoricone |
| PTPN11 | gancao | liquiritin |
| HDAC3 | gancao | liquiritin |
| BMP4 | gancao | Lupiwighteone |
| ASAH1 | gancao | Mairin |
| CHEK1 | gancao | Medicarpin |
| CXCR4 | gancao | naringenin |
| HTR2C | gancao | naringenin |
| DRD5 | gancao | naringenin |
| MME | gancao | Odoratin |
| PGR | gancao | Phaseol |
| ESR1 | gancao | Phaseolinisoflavan |
| INSR | gancao | Phaseolinisoflavan |
| MAPK8 | gancao | Phaseolinisoflavan |
| CA4 | gancao | Phaseolinisoflavan |
| APP | gancao | Phaseolinisoflavan |
| RXRB | gancao | Phaseolinisoflavan |
| SIRT1 | gancao | Phaseolinisoflavan |
| NCOA2 | gancao | Phaseolinisoflavan |
| CYP11B2 | gancao | quercetin |
| SRD5A2 | gancao | quercetin |
| TBXA2R | gancao | quercetin |
| PPARG | gancao | quercetin |
| HDAC1 | gancao | quercetin |
| ADRB1 | gancao | Semilicoisoflavone B |
| JAK2 | gancao | shinpterocarpin |
| PDE4D | gancao | shinpterocarpin |
| LRRK2 | gancao | shinpterocarpin |
| CA3 | gancao | shinpterocarpin |
| P2RY1 | gancao | shinpterocarpin |
| GRIN1 | gancao | Sigmoidin-B |
| CCR8 | gancao | Sigmoidin-B |
| CXCR2 | gancao | Sigmoidin-B |
| CASP1 | gancao | Sigmoidin-B |
| P2RY12 | gancao | Vestitol |
| F2RL3 | gancao | Vestitol |
| NOS3 | gancao | Vestitol |
| RET | gancao | Vestitol |
| F9 | gancao | Vestitol |
| DUSP3 | gancao | Vestitol |
| ODC1 | gancao | Vestitol |
| MTNR1A | gancao | Vestitol |
| AKR1C1 | guizhi | Cinnamic acid |
| AKR1B1 | guizhi | Cinnamic acid |
| CA9 | guizhi | Cinnamic acid |
| APOB | guizhi | taxifolin |
| CHRM1 | guizhi | Tetradecanal |
| ADA | guizhi | Tetradecanal |
| AR | guizhi | Tetradecanal |
| CYP1B1 | shaoyao | (3S,5R,8R,9R,10S,14S)-3,17-dihydroxy-4,4,8,10,14-pentamethyl-2,3,5,6,7,9-hexahydro-1H-cyclopenta[a]phenanthrene-15,16-dione |
| CXCR1 | shaoyao | albiflorin_qt |
| CRHR1 | shaoyao | albiflorin_qt |
| ACP1 | shaoyao | albiflorin_qt |
| AGTR1 | shaoyao | albiflorin_qt |
| BTK | shaoyao | albiflorin_qt |
| ABL1 | shaoyao | albiflorin_qt |
| ADAM17 | shaoyao | albiflorin_qt |
| CYP3A4 | shaoyao | kaempferol |
| CES1 | shaoyao | kaempferol |
| ADRA1D | shaoyao | kaempferol |
| MAOA | shaoyao | kaempferol |
| AKR1C2 | shaoyao | kaempferol |
| AKR1C3 | shaoyao | kaempferol |
| ALOX5 | shaoyao | kaempferol |
| CASP8 | shaoyao | kaempferol |
| GSR | shaoyao | kaempferol |
| ABCC1 | shaoyao | kaempferol |
| APEX1 | shaoyao | kaempferol |
| ATM | shaoyao | kaempferol |
| CDK7 | shaoyao | kaempferol |
| DRD1 | shaoyao | kaempferol |
| AXL | shaoyao | kaempferol |
| ABCB1 | shaoyao | Lactiflorin |
| ADRA2A | shaoyao | Lactiflorin |
| CHRM3 | shaoyao | Lactiflorin |
| ADORA2A | shaoyao | Lactiflorin |
| ADRA2C | shaoyao | Lactiflorin |
| CCR3 | shaoyao | Lactiflorin |
| CCR4 | shaoyao | Lactiflorin |
| CALCA | shaoyao | Lactiflorin |
| ADORA1 | shaoyao | Mairin |
| ALDH2 | shaoyao | Mairin |
| AKT1 | shaoyao | Mairin |
| ADRA1B | shaoyao | paeoniflorin |
| ADCY10 | shaoyao | paeoniflorin |
| AMY1A | shaoyao | paeoniflorin |
| BCHE | shaoyao | Paeonol |
| F7 | shengjiang | curcumin |
| AVPR1A | shengjiang | curcumin |
| PRKG2 | shengjiang | curcumin |
| OPRK1 | shengjiang | curcumin |
| TGM2 | shengjiang | curcumin |
| RAF1 | shengjiang | curcumin |
| S1PR1 | shengjiang | curcumin |
| HAS2 | shengjiang | curcumin |
| FCER2 | shengjiang | Dihydrocapsaicin |
| JUN | shengjiang | Dihydrocapsaicin |
| NPY1R | shengjiang | Dihydrocapsaicin |
| ICAM1 | shengjiang | gingerol |
| CAT | shengjiang | gingerol |
| FGFR1 | shengjiang | gingerol |
| EIF2AK2 | shengjiang | gingerol |
| BRD2 | shengjiang | gingerol |
| KCNJ1 | shengjiang | gingerol |
| LCK | shengjiang | gingerol |
| NCSTN | shengjiang | poriferast-5-en-3beta-ol |
| SLC6A11 | shengjiang | poriferast-5-en-3beta-ol |
| SCD | shengjiang | poriferast-5-en-3beta-ol |
