# Supplementary material for: To Predict Anti-Inflammatory and Immunomodulatory Targets of Guizhi Decoction in Treating Asthma Based on Network Pharmacology, Molecular Docking, and Experimental Validation
Source: Evid Based Complement Alternat Med. 2021 Dec 20;2021:9033842. doi: 10.1155/2021/9033842 (PMC8712140; doi:10.1155/2021/9033842)
Supplement: Supplementary Materials — Supplementary Material Table S1: 134 active compounds from TCMSP database and literature in Guizhi Decoction. Supplementary Material Table S2: drug targets information of different ingredients in Guizhi Decoction. Supplementary Material Table S3: target information at the intersection of drug targets and disease targets. Supplementary Material Table S4: core gene information filtered according to the “betweenness,” “closeness,” and “degree” values. Supplementary Material Table S5: details of the known ligand of the top targets. [file 9033842.f1.zip › 9033842.f1/Supplementary Material Table S4 (1).docx]

| ***Supplementary Material***  **Table S4** Core gene information filtered according to the "Betweenness", "Closeness" and "Degree" values | | | |
| --- | --- | --- | --- |
| **Gene** | **Betweenness** | **Closeness** | **Degree** |
| NFKB1 | 96.94599 | 0.574074 | 25 |
| AGTR1 | 69.87495 | 0.519553 | 21 |
| MAPK1 | 268.5666 | 0.632653 | 40 |
| HTR2A | 0 | 0.458128 | 15 |
| FGR | 40.25721 | 0.531429 | 16 |
| RELA | 126.7549 | 0.58125 | 27 |
| AKT1 | 304.8071 | 0.62 | 37 |
| LYN | 125.4269 | 0.58125 | 30 |
| FYN | 179.4147 | 0.603896 | 34 |
| TBXA2R | 137.5665 | 0.510989 | 20 |
| LCK | 116.018 | 0.592357 | 33 |
| HDAC5 | 5.438093 | 0.415179 | 3 |
| PDGFRB | 41.69395 | 0.534483 | 22 |
| KDR | 5.782405 | 0.458128 | 12 |
| EDNRB | 22.48215 | 0.489474 | 16 |
| PTAFR | 56.92633 | 0.516667 | 17 |
| MAPK14 | 186.6195 | 0.547059 | 25 |
| NRAS | 82.23013 | 0.560241 | 29 |
| CCND1 | 20.37648 | 0.476923 | 12 |
| ABL1 | 3.643493 | 0.447115 | 9 |
| KIT | 3.398156 | 0.481865 | 11 |
| JAK2 | 76.48646 | 0.574074 | 26 |
| TNF | 579.2385 | 0.628378 | 38 |
| EDNRA | 130.134 | 0.556886 | 20 |
| MAPK8 | 178.6909 | 0.592357 | 30 |
| PRKCA | 240.5296 | 0.574074 | 25 |
| ADRBK1 | 127.2531 | 0.513812 | 24 |
| EP300 | 209.4665 | 0.516667 | 22 |
| F2 | 175.8272 | 0.553571 | 25 |
| PRKCE | 76.06811 | 0.528409 | 16 |
| ERBB2 | 5.694314 | 0.481865 | 14 |
| ADRA1B | 12.1176 | 0.47449 | 19 |
| VEGFA | 134.3648 | 0.57764 | 25 |
| CHRM1 | 107.8121 | 0.537572 | 22 |
| ITGB3 | 29.76824 | 0.489474 | 20 |
| P2RY1 | 22.48215 | 0.489474 | 16 |
| HIF1A | 6.862159 | 0.479381 | 11 |
| TGFB1 | 45.43829 | 0.537572 | 15 |
| IKBKB | 9.771127 | 0.447115 | 12 |
| HSPA8 | 48.23222 | 0.497326 | 9 |
| SYK | 43.6899 | 0.508197 | 18 |
| HRAS | 234.0602 | 0.611842 | 36 |
| CXCR4 | 236.7989 | 0.563636 | 28 |
| ESR1 | 17.21187 | 0.481865 | 15 |
| PRKCQ | 69.48754 | 0.531429 | 16 |
| CCR1 | 15.85863 | 0.497326 | 18 |
| RHOA | 99.14615 | 0.592357 | 31 |
| F2RL3 | 17.13305 | 0.494681 | 16 |
| HSP90AA1 | 142.9223 | 0.553571 | 29 |
| PTK2 | 63.77199 | 0.560241 | 24 |
| EGFR | 293.7313 | 0.596154 | 31 |
| CCR3 | 25.30503 | 0.479381 | 22 |
| HSP90AB1 | 8.798903 | 0.469697 | 11 |
| S1PR1 | 314.8142 | 0.584906 | 29 |
| MMP9 | 1.769292 | 0.449275 | 6 |
| BCL2 | 10.53338 | 0.467337 | 10 |
| PRKCZ | 63.85649 | 0.525424 | 22 |
| CXCR1 | 41.96981 | 0.489474 | 20 |
| ITGAV | 16.8943 | 0.472081 | 16 |
| AR | 20.53571 | 0.479381 | 12 |
| CSK | 8.468402 | 0.486911 | 17 |
| ADRA2C | 42.79641 | 0.494681 | 20 |
| JUN | 121.4422 | 0.540698 | 21 |
| DRD2 | 3.59795 | 0.462687 | 18 |
| ADRA2B | 42.79641 | 0.494681 | 20 |
| CHUK | 9.771127 | 0.447115 | 12 |
| MTOR | 13.68713 | 0.481865 | 11 |
| ADRA2A | 42.79641 | 0.494681 | 20 |
| PIK3CG | 12.70081 | 0.502703 | 12 |
| BDKRB2 | 89.59527 | 0.510989 | 30 |
| PDPK1 | 33.92491 | 0.489474 | 18 |
| FGF2 | 13.15208 | 0.481865 | 15 |
| JAK1 | 37.31755 | 0.525424 | 22 |
| PTPN11 | 50.83239 | 0.560241 | 26 |
| CASR | 141.1294 | 0.547059 | 31 |
| CHRM2 | 70.89852 | 0.505435 | 21 |
| FPR1 | 80.6384 | 0.537572 | 24 |
| APP | 579.2385 | 0.628378 | 38 |
| ADORA1 | 0 | 0.458128 | 17 |
| OPRM1 | 49.51907 | 0.508197 | 21 |
| STAT6 | 69.58028 | 0.508197 | 16 |
| ADRB2 | 62.10931 | 0.469697 | 13 |
| GCGR | 46.23745 | 0.465 | 17 |
| STAT3 | 505.1282 | 0.628378 | 38 |
| NR3C1 | 26.53182 | 0.479381 | 16 |
| PTGDR | 6.489974 | 0.400862 | 3 |
| MAPK3 | 284.5592 | 0.636986 | 41 |
| HTR1A | 0 | 0.458128 | 17 |
| RARA | 3.874293 | 0.465 | 8 |
| RAF1 | 15.76501 | 0.525424 | 18 |
| OPRD1 | 40.8232 | 0.502703 | 20 |
| CASP8 | 43.22307 | 0.510989 | 12 |
| SRC | 364.3563 | 0.645833 | 43 |
| IL2 | 579.2385 | 0.628378 | 38 |
