# Supplementary material for: To Predict Anti-Inflammatory and Immunomodulatory Targets of Guizhi Decoction in Treating Asthma Based on Network Pharmacology, Molecular Docking, and Experimental Validation
Source: Evid Based Complement Alternat Med. 2021 Dec 20;2021:9033842. doi: 10.1155/2021/9033842 (PMC8712140; doi:10.1155/2021/9033842)
Supplement: Supplementary Materials — Supplementary Material Table S1: 134 active compounds from TCMSP database and literature in Guizhi Decoction. Supplementary Material Table S2: drug targets information of different ingredients in Guizhi Decoction. Supplementary Material Table S3: target information at the intersection of drug targets and disease targets. Supplementary Material Table S4: core gene information filtered according to the “betweenness,” “closeness,” and “degree” values. Supplementary Material Table S5: details of the known ligand of the top targets. [file 9033842.f1.zip › 9033842.f1/Supplementary Material Table S5.docx]

***Supplementary Material***

**Table S5** Details of the known ligand of the top targets

| **The known ligand** | **Target** | **PubChem CID** |
| --- | --- | --- |
| EKO | MAPK1 | 2767228 |
| 5ID | MAPK3 | 97297 |
| KSA | NFKB1 | 3035817 |
| 17V | PIK3CG | 57900314 |
| KSA | RELA | 3035817 |
| SM5 | RAF1 | 11653652 |
| KSA | IKBKB | 3035817 |
| GNP | HRAS | 135403657 |
| KSA | CHUK | 3035817 |
